# The need for further guidance on the handling of multiple outcomes in randomized controlled trials: a scoping review of the methodological literature

**DOI:** 10.1016/j.jclinepi.2025.111724

**Published:** 2025-05

**Authors:** Hadeel Hussein, Rod S. Taylor, Anthony Muchai Manyara, Anthony Purvis, Richard Emsley, Rui Duarte, Valerie Wells, Yimin Jiang, Grace O. Dibben

**Affiliations:** a MRC/CSO Social and Public Health Sciences Unit, School of Health and Wellbeing, University of Glasgow, Glasgow, United Kingdom; b Robertson Centre for Biostatistics, School of Health and Wellbeing, University of Glasgow, Glasgow, United Kingdom; c Global Health and Ageing Research Unit, Bristol Medical School, University of Bristol, Bristol, United Kingdom; d Department of Biostatistics and Health Informatics, Institute of Psychiatry, Psychology & Neuroscience (IoPPN), King's College London, London, United Kingdom; e Department of Health Data Science, University of Liverpool, Liverpool, United Kingdom; f Saluda Medical Pty Ltd, Artarmon, New South Wales, Australia; g Nottingham Clinical Trials Unit, University of Nottingham, Nottingham, United Kingdom

**Keywords:** Multiple outcomes, Multiple endpoints, Multiplicity, Composite outcome, Randomized clinical trial, Complex intervention

## Abstract

**Objectives:**

To review current methodological guidance for handling and reporting of multiple outcomes (MOCs) in randomized controlled trials (RCTs).

**Study Design and Setting:**

A scoping review with bibliographic database searches including Embase, PubMed, and Web of Science up to January 16, 2025 was conducted. Inclusion criteria were articles that: (1) provide advice on the design, analysis, or reporting of RCTs using MOCs; and/or (2) discuss statistical approaches for handling MOCs in RCTs. Six specific websites were also checked for formal and reporting guidelines. Included articles were summarized using thematic analysis.

**Results:**

Searches retrieved 1716 articles of which 123 were included with additional 25 articles from updated search. Eight additional articles were identified by the specific website search. Six main subthemes on methodological recommendations for using MOCs were identified from 74 of 123 articles (60%): (1) need to prespecify outcomes and analysis, (2) multiplicity adjustment, (3) power and sample size implications, (4) secondary outcomes multiplicity, (5) considerations of MOCs correlation, and (6) specific applications of MOCs. Recommendations on coprimary and composite outcomes were also identified, including their features, analyses methods, reporting, and challenges. Statistical methods for analyzing MOCs were discussed in 53 of 123 articles (43%), with the majority describing modifications of pre-existing statistical approaches.

**Conclusion:**

Current recommendations on using MOCs in RCTs focus primarily on statistical considerations and trials of licensing drugs or medical devices. Areas for further methodological research and guidance include reporting of the rationale for the use and selection of MOCs in RCTs and considerations for trials undertaken in nonregulatory setting, including complex interventions.

## Highlights

• Focus of multiple outcomes guidance is the statistical issues in regulatory trials.
• Absence of wider methods/reporting considerations for multiple outcomes in trials.
• Limited guidance on approaching and reporting of secondary outcomes multiplicity.

## Graphical abstract

**Figure:**
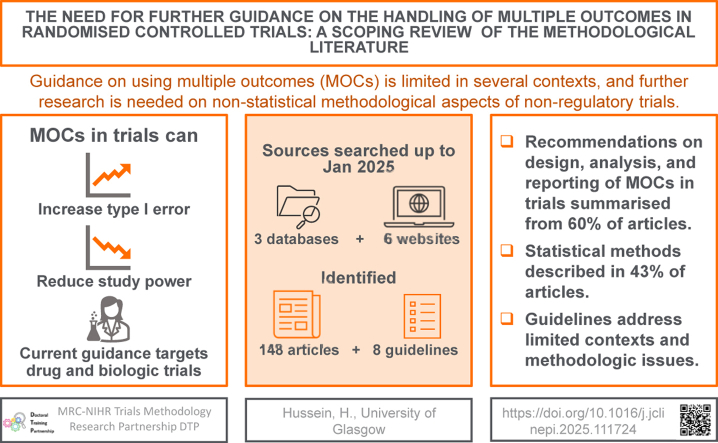

**Plain Language Summary**

**Why do we need multiple outcomes (MOCs) in medical research?**

New treatments or health interventions are usually assessed by a form of medical experiments. These are called randomized controlled trials RCTs. In these experiments, or RCTs, the effect of the new treatment is compared to other available practices. The effect under assessment is reflected by an “outcome.” Because these treatments often have several potential effects on our health, researchers would be interested in assessing MOCs.

**What is the problem?**

When we run multiple tests to assess MOCs, there is an increased risk of concluding positive treatment effects by chance. This diminishes the trustworthiness of RCTs results. The medical literature provides some guidance on how researchers can design their RCTs and test their MOCs more reliably. However, this guidance can be limited to certain situations where the aim is for marketing approval of the new treatment. An example is assessing a new drug treatment.

**How did we conduct our review?**

We aimed to examine the literature on the guidance for using MOCs in RCTs. In addition, we aimed to identify areas for further research to improve the use of MOCs. We searched medical literature sources and the webpages of guidance-producing bodies up to January 2025.

**What are our key findings?**

We report our findings from 123 scientific articles and eight guidance reports. A key finding is that the available guidance focuses on the setting described above, the regulatory setting. In addition, these recommendations address certain issues of MOCs that are “statistical” in nature. Furthermore, we discuss this limitation and the need for a broader focus on wider nonstatistical problems in nonregulatory situations where researchers are interested in assessing MOCs.

Our review highlights the opportunities to complete gaps in guidance on the use of MOCs. This in turn can assist future production of more reliable results from RCTs to accurately inform the potential users of new treatments.

**What is new?**

**Key findings**
• Focus of multiple outcomes guidance is the statistical issue in regulatory trials.

**What this adds to what is known?**
• Absence of wider methods/reporting considerations for multiple outcomes in trials.
• Limited guidance on approaching and reporting of secondary outcomes’ multiplicity.

**What is the implication and what should change now?**
• Further research is needed to improve the handling of multiple outcomes in randomized controlled trials.

## 1 Introduction

### 1.1 Background

It is recommended that randomized controlled trials (RCTs) prespecify a single primary outcome [1,2]. However, treatments can have various impacts on patients' health, especially in complex interventions (CI) trials [3]. Consequently, researchers often seek to assess multiple outcomes (MOCs) to holistically evaluate participants’ responses [4,5]. However, if MOCs are not accounted for appropriately in the analysis of RCTs, the probability of obtaining statistically significant results by chance may increase—so-called type I (or α-level) error [4,[6], [7], [8], [9]]. This multiplicity raises challenges for the design, analysis, and reporting of RCTs and has been identified as a priority for methodological research [10].

Several approaches have been proposed to address MOCs in RCTs, including the use of coprimary outcomes, where statistically significant treatment effect on each outcome is required to prove treatment efficacy; multiplicity testing procedure (MTP) to adjust the α-level, such as the Bonferroni method, or global testing methods where a single test is performed across multiple-related outcomes, such as O'Brien's and Hotelling's T^2^ tests [[11], [12], [13]]. Composite outcomes are another approach, such as the major adverse cardiovascular events (MACE) [14].

Despite the frequent use of MOCs in RCTs and the availability of statistical approaches to account for multiplicity, studies have shown that appropriate data analyses and reporting practices are not consistently implemented. In 2015, Vickerstaff et al reviewed 209 RCTs and found that more than 60% of 67 RCTs with MOCs did not account for primary outcomes multiplicity appropriately [8]. Furthermore, separate testing of secondary outcomes was reported in 136 trials of this review, of which about 85% did not account for multiplicity [8].

Guidance on handling MOCs in trials and reporting guidelines have been published by US Food and Drug Administration (FDA) [4], European Network for Health Technology Assessment (EUnetHTA) [15], European Medicines Agency (EMA) [16], The International Council for Harmonisation of Technical Requirements for Pharmaceuticals for Human Use (ICH) [2], and the Outcomes extensions from the Standard Protocol Items: Recommendations for Interventional Trials (SPIRIT) [17] and Consolidated Standards for Reporting Trials (CONSORT) [18]. The aims of this study were to undertake a contemporary examination of handling MOCs in RCTs, to map the available guidance, and to identify gaps where further research can inform the understanding of the methodological complexities of MOCs.

### 1.2 Objectives

Specific research objectives were:

1. To identify the methodological guidance for using MOCs in RCTs.
2. To identify common approaches, including novel statistical methods, used to address multiplicity of MOCs in RCTs.
3. To identify guidance that specifically addresses secondary outcome multiplicity and the use of MOCs in CI trials.

## 2 Methods

This scoping review broadly followed the principles of the Joanna Briggs Institute (JBI) framework for scoping reviews [19]. The scoping review protocol [20] was preregistered (https://osf.io/gnwk8/) and the Preferred Reporting Items for Systematic Reviews and Meta-Analyses statement for scoping reviews (PRISMA-ScR, Supplementary file 1) is used for reporting [21].

### 2.1 Eligibility criteria

Articles were included that: (1) provide advice/guidance on the use of MOCs in the context of the design, analysis, or reporting of RCTs; or (2) discuss statistical approaches for the handling of MOCs in RCTs. Articles were excluded that focused on the use of MOCs in meta-analyses, a non-RCT setting, multiple comparisons without a focus on MOCs, not available as full text, or were not published in English language.

### 2.2 Search process

Bibliographic databases (Embase, PubMed, Web of Science) were searched from inception up to July 22, 2022 using a mix of text terms and MeSH headings (“multiple outcome(s)” OR “multiple comparison(s)” OR “multiplicity” OR “composite outcome(s)”) and “clinical trials.” The search strategy (Supplementary file 2) was designed in consultation with an experienced information specialist (V.W.). Specific websites were also searched, including Enhancing the QUAlity and Transparency Of health Research (EQUATOR), ICH, FDA, EMA, EUnetHTA, and the Medicines and Healthcare Products Regulatory Agency (MHRA). Study selection was conducted by two independent reviewers across a pool of 6 reviewers (H.H., G.O.D., A.P., R.S.T., A.M., Y.J.).

An updated search was conducted from July 22, 2022, to January 16, 2025, in the same databases following the same eligibility criteria.

### 2.3 Data extraction

The data extraction template was predesigned and piloted. Descriptive data from included studies were extracted, including year and country of publication, type of publication, and research area alongside guidance/advice on the use of MOCs and statistical analysis for MOCs. Specific considerations of MOCs for CI were noted. Independent data extraction was performed by three reviewers (H.H., A.P., Y.J.) and approximately 25% cross-checked by one of four reviewers (H.H., A.P., Y.J., R.S.T.). Discrepancies were resolved by discussion among the research group.

### 2.4 Data synthesis

Descriptive and thematic analyses were conducted in Microsoft Excel. One reviewer (H.H.) descriptively analyzed the articles characteristics using counts and percentages. Guidance was thematically analyzed, for which the major themes (design, analysis, reporting, and challenges) were predetermined. Three reviewers (H.H., R.S.T., and A.M.) met twice to identify and agree on relevant subthemes and generated codes. Articles describing statistical methods were categorized into new or modified methods by reviewer consensus (H.H., G.O.D., R.S.T., and A.P.), with one reviewer (H.H.) summarizing its impact on type I and II errors and outcomes correlation.

## 3 Results

### 3.1 Search results

**Figure 1:**
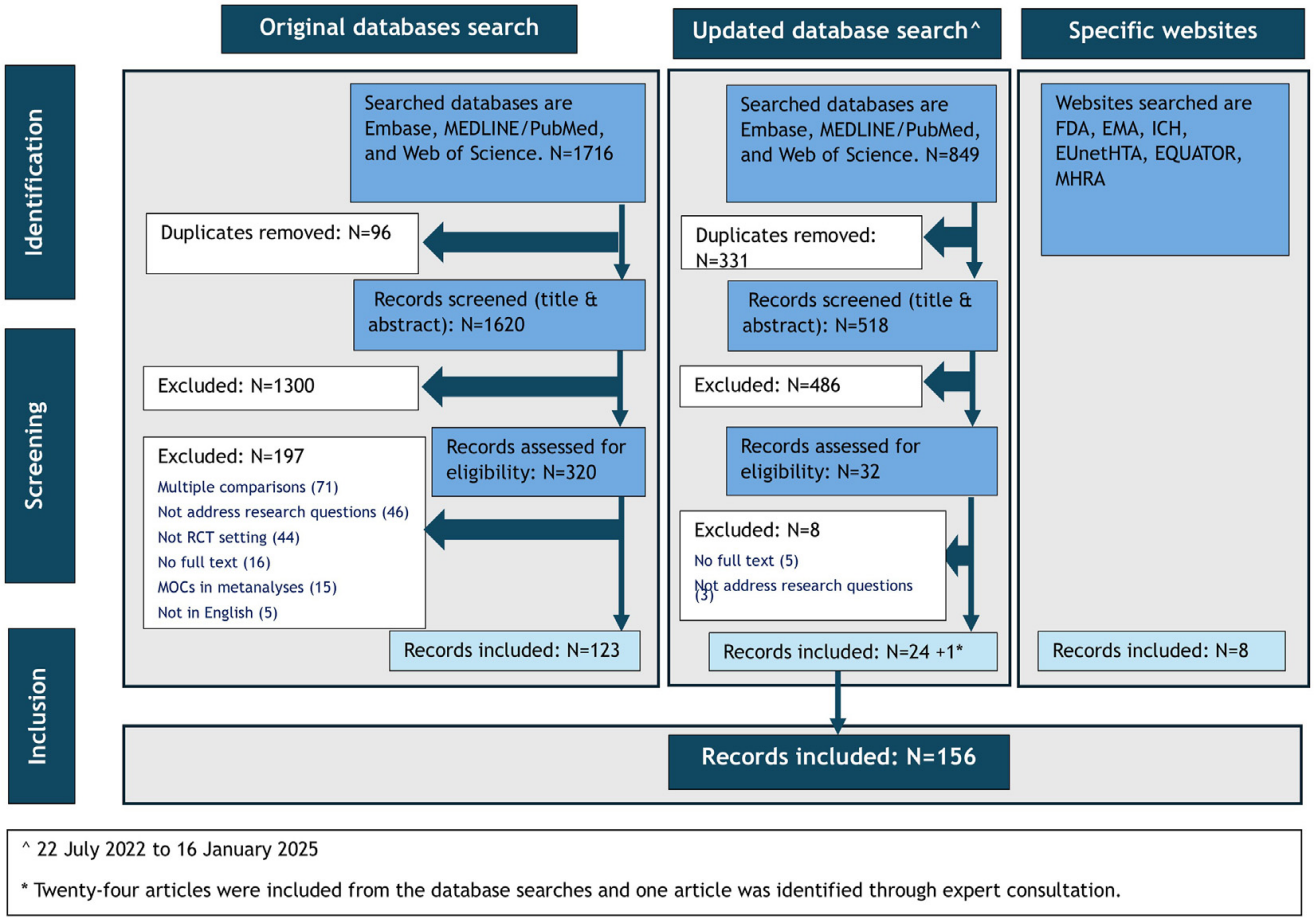
PRISMA diagram illustrating the search process and the number of screened, excluded, and included articles. PRISMA, Preferred Reporting Items for Systematic Reviews and Meta-Analyses; FDA, The US Food and Drug Agency; EMA, European Medicines Agency; ICH, The International Council for Harmonisation of Technical Requirements for Pharmaceuticals for Human Use; EUnetHTA, European Network for Health Technology Assessment; EQUATOR, Enhancing the QUAlity and Transparency Of health Research; MHRA, Medicines and Healthcare Products Regulatory Agency; MOCs, multiple outcomes; RCT, randomized controlled trial.

Original database searches retrieved a total of 1716 articles, from which 123 articles were included (Fig 1). An additional eight records were included from websites of regulatory bodies and reporting guidelines. Reference list of all included articles is provided in Supplementary file 3^S1-S131^.

The updated searches retrieved 849 records from which 24 were included with one additional article identified through expert consultation. Thirteen papers discussed themes aligned to the guidance discussed below, four reported special applications, and two discussed challenges. Additionally, 13 articles presented innovative statistical procedures for MOCs (all modified from pre-existing methods). Supplementary file 8 provides a list of these included references^S8-1-S8-26^ with summary of their characteristics, focus, and the statistical methods identified.

The results reported under section 3.2 onward reflect findings from the original search results only.

### 3.2 Characteristics of the included articles

Table 1 summarizes the characteristics of the 123 articles in this scoping review with detailed description provided in Supplementary file 4. Most articles were research publications (*N* = 113, 92%), predominantly published between 2011 and 2020 (*N* = 48, 39%). Lead authors were most frequently affiliated to an academic institute (*N* = 86, 70%) and based in North America (*N* = 88, 71.5%) or Europe (*N* = 36, 29%). Among the articles, 74 (60%) provided general guidance, and 53 (43%) described statistical methods.^1^ Thirty-three (27%) articles focused on specific trial designs while 18 (15%) focused on specific clinical areas. Of the eight articles from regulatory and guidelines websites, four (50%) had recommendations on handling MOCs in drug and/or health technology assessment trials (HTA) and five on trials reporting (62%).

**Table 1:** Characteristics of the included articles (*N* = 123)

Characteristics	Number of articles (%)
Publication year	
1981–1990	3 (2%)
1991–2000	27 (22%)
2001–2010	34 (28%)
2011–2020	48 (39%)
2021–2022	11 (9%)
Regions of publication first authors	
North America	85 (69%)
Europe	33 (27%)
Asia	4 (3%)
Australia	1 (1%)
Affiliation of first authors	
Academic	86 (70%)
Industrial	21 (17%)
Regulatory	15 (12%)
Journal associate editor	1 (1%)
Funding	
Funding not reported	65 (53%)
Public/governmental agency	32 (26%)
Mixed funding sources	11 (9%)
Pharmaceutical company	4 (3%)
Academic	3 (2%)
Regulatory agency	3 (2%)
No funding received	3 (2%)
Nonprofit organization	2 (2%)
Publication type	
Journal publication	113 (92%)
Other	10 (8%)
Research area^a^	
General MOCs^b^ guidance	74 (60%)
Statistical method for MOCs	53 (43%)

a There is an overlap between the categories under this characteristic as four articles provided both general guidance and statistical method description.

b MOCs, multiple outcomes.

### 3.3 Summary of regulatory and trial reporting guidance

Identified methodological guidance from leading regulatory agencies, FDA, EMA, ICH, and EUnetHTA [2,4,15,16] and reporting guidance from SPIRIT and CONSORT guidelines and EUnetHTA, is summarized in Table 2 [15,17,18,22,23].

**Table 2:** Summary of formal guidance identified from regulatory bodies and reporting guidelines

Summary item	ICH [2]	EMA [16]	FDA^a^ [4]	EUnetHTA [15]	CONSORT (core) [23]	SPIRIT (core) [22]	SPIRIT-outcomes [17]	CONSORT-outcomes [18]
Clearly define and prespecify outcomes and prespecify methods to be used for multiplicity adjustment.	**Y**	**Y**	**Y**	**Y**	**Y**	**Y**	**Y**	**Y**
Prespecify hypotheses, detailed statistical analyses plan, and impact on type I error.	**Y**			**Y**				
Reporting where multiplicity adjustment was not done (with justification).	**Y**			**Y**			**Y**	**Y**
No adjustment where coprimary outcomes are used. However, power and sample size will be negatively impacted by this approach, therefore requires careful planning.	**Y**	**Y**	**Y**					
Composite outcomes should be defined, justified, and a full definition of its component provided.							**Y**	**Y**
Composite outcomes features, advantages, or disadvantages.	**Y**	**Y**	**Y**				**Y**	**Y**
Algorithm for combining composite components should be predefined.	**Y**						**Y**	**Y**
Secondary analyses for components are required to aid interpretation of clinically meaningful results on a composite.	**Y**	**Y**	**Y**					
Adjust for multiplicity on composite components analyses where the purpose is to provide confirmatory conclusions; otherwise, clinical judgment can be used to interpret results.			**Y**					
Secondary outcomes are to be kept to a minimum and be prespecified.	**Y**	**Y**	**Y**					
The analyses methods for secondary outcomes, including any MTPs where applicable, should be reported in protocols and in final RCT reports.							**Y**	**Y**
Secondary outcomes can be tested for confirmatory conclusions if it is part of a predefined MTP plan and is tested after achieving significance on the primary outcome(s).		**Y**	**Y**					
Where sample size is not based on primary outcome, this should be reported and justified.	**Y**							
Controlling type I error increase type II error, hence larger sample size is needed to accommodate this loss of power.			**Y**					
Power can be calculated for outcomes other than the primary, or else, where calculations were based on single outcome the available level of power for other outcomes should be reported.						**Y**		
Avoid unplanned/post hoc analyses as these can introduce bias, multiplicity concerns, and lead to data dredging.	**Y**	**Y**	**Y**	**Y**				

Y indicates that an item was addressed in the relevant guidance.

a FDA, The US Food and Drug Agency; EMA, European Medicines Agency; ICH, The International Council for Harmonisation of Technical Requirements for Pharmaceuticals for Human Use; EUnetHTA, European Network for Health Technology Assessment; SPIRIT, Standard Protocol Items: Recommendations for Interventional Trials; CONSORT, Consolidated Standards for Reporting Trials.

A detailed summary of the FDA 2022 guidance is provided in Supplementary file 5. In addition, the guidance extracted from the included articles is mapped against the recommendations from the FDA 2022 (Supplementary file 6). In summary, among 96 items under the identified subthemes, 22 (23%) were addressed by the FDA 2022 guidance, 19 (20%) provide further elaborations, and 52 (54%) were not addressed. The latter mainly covered MOCs correlation, power and sample size implications, reporting, challenges, or discuss specific MTPs. Three of the subthemes discussed specific applications for MOCs, including coprimary and composite outcomes, hence items under these subthemes were not relevant to this mapping.

### 3.4 Synthesis of the guidance

We categorize guidance from 74 articles under two distinct subheadings: (1) general guidance for the use of MOCs and (2) guidance on the use of specific approaches for handling MOCs. Within each, we consider the implications for trial design and reporting. Challenges of handling MOCs are summarized from the 123 articles.

#### 3.4.1 General guidance for the use of MOCs

##### 3.4.1.1 Trial design

Six subthemes were identified on general guidance for the design of trials with MOCs: (1) prespecification, (2) indications for multiplicity adjustment, (3) study power and sample size, (4) secondary outcomes multiplicity, (5) MOCs correlation (ie, degree of dependence between MOCs and its implications on choice of MTPs), and (6) MOCs in specific settings.

“Prespecification” “was discussed in 21 of the 74 articles (28%). Articles recommended prespecifying research questions and hypotheses (*N* = 10/21, 48%),^S1,S2,S15,S17,S40,S63,S71,S86,S99,S100^ outcomes classification (primary, secondary, or exploratory) and definitions (*N* = 11/21, 52%),^S14,S15,S17,S19,S65,S68,S78,S80,S83,S86,S99^ and the clinical decision rule with the outcomes relevant to it (*N* = 6/21, 29%).^S15,S43,S71,S78,S99,S101^ A clinical decision rule is the rule of which and how many of the defined outcomes affected by a treatment under study would prove the treatment efficacy.^S15^ In addition, prespecification of the need for multiplicity adjustment, chosen procedures, and rationale should be prespecified (*N* = 9/21, 43%).^S5,S19,S68,S71,S78,S86,S99-101^ Where exploratory outcomes are used, these should be clearly prespecified and labeled as such (*N* = 2/21, 9.5%).^S59,S99^

**Figure 2:**
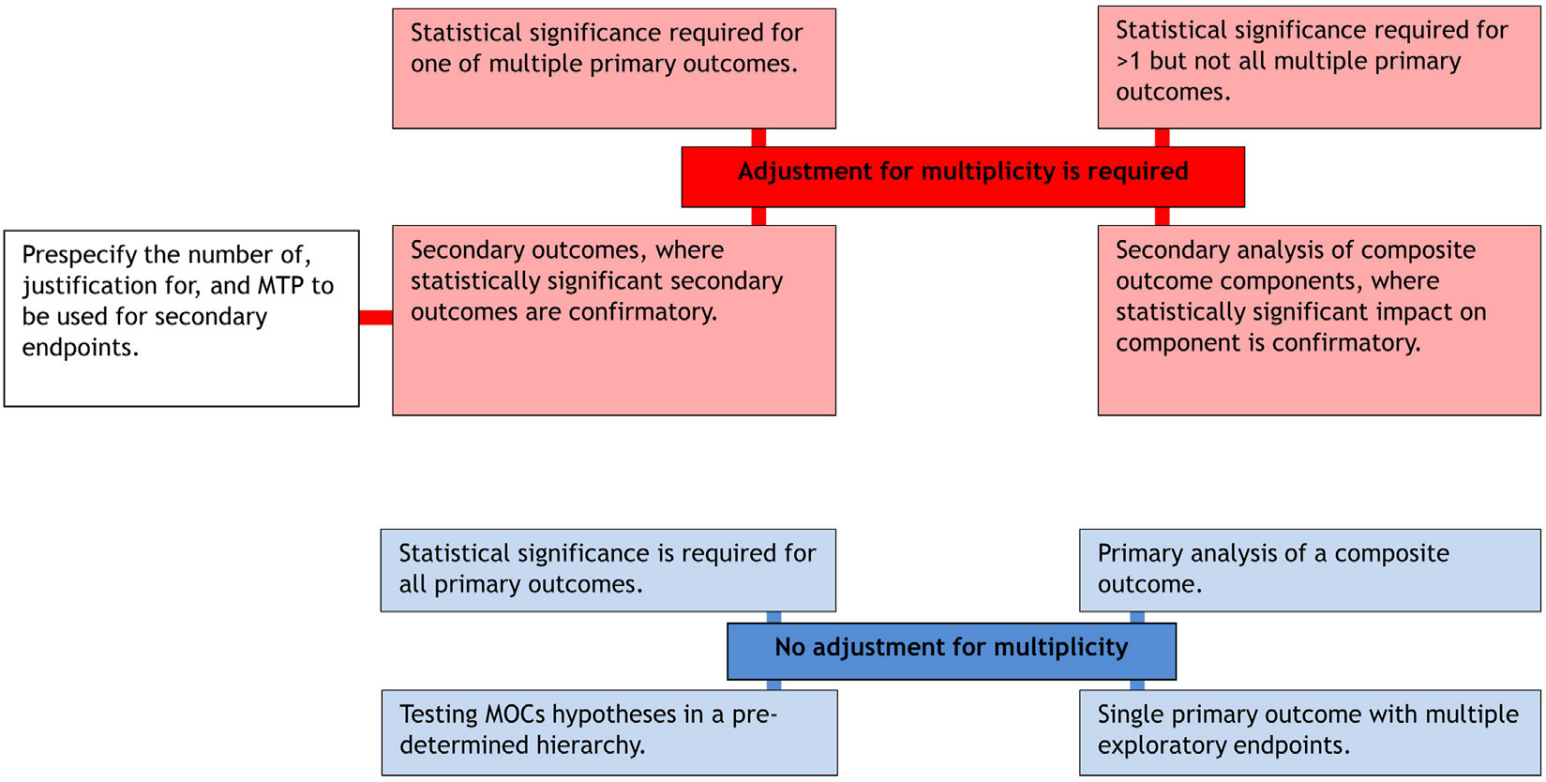
Situations where multiplicity adjustment is required (red-upper) vs. not required (blue-lower) as summarized from the reviewed articles. MOCs, multiple outcomes.

Multiplicity adjustment and its indications were discussed in 23 of the 74 articles (31%). Of these articles, seven of the 23 articles recommend strongly controlling type I error (29%).^S2,S5,S19,S34,S65,S99,S100^ Two of the 23 articles (8%) suggested to focus on the terminology used to describe groups of outcomes (primary vs. secondary) and the need for agreement among stakeholders on terminology to avoid confusion on the need to apply an MTP. ^S78,S80^ Four articles of 23 (17%) discussed the importance of bringing the false positive rate to a desired level to avoid the impact of MTPs on type II error, known as the False Discovery Rate (FDR).^S59,S71,S76,S99^ Figure 2 summarizes the recommendations of 18 out of the 74 (24%) articles regarding when multiplicity adjustment is indicated or not.^S5,S15,S21,S34,S56,S59,S64,S71,S76,S79,S80,S83,S84,S99,S101,S102,S110,S117^

**Figure 3:**
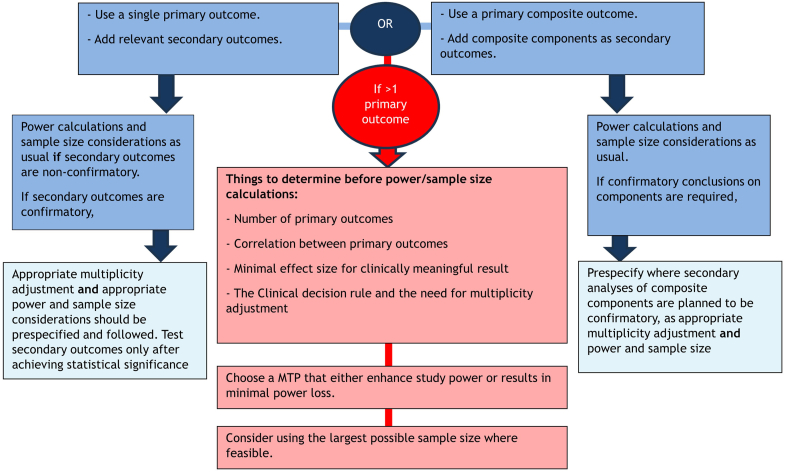
Study power and sample size considerations when using MOCs (red-central arrow) vs. when using single primary or a composite outcome as summarized from the reviewed articles. MOCs, multiple outcomes. (For interpretation of the references to colour in this figure legend, the reader is referred to the web version of this article.)

Considerations of study power and sample size when using MTPs were explored in 29 of the 74 articles (39%). Multiplicity adjustments can increase type II error, leading to loss of power and higher sample size requirements, as noted in four of 29 articles (14%).^S5,S19,S59,S76^ Another three articles (10%) highlighted that the extent of this impact depends on the number of primary outcomes and the correlation among these.^S2,S71,S99^ Fourteen of 29 articles (48%) further discussed the impact of certain MTPs on power such as having power gains or avoiding loss of power.^S1,S2,S5,S33,S35,S38,S48,S65,S68,S70,S79,S80,S99,S102^ Figure 3 summarizes the implications of using MOCs on study power and sample size compared to using a single primary or composite outcome from 24/74 (32%) articles.^S5,S17,S19,S21,S23,S33-S35,S40,S56,S59,S64-S66,S68,S71,S76,S77,S79,S80,S98,S99,S101,S110^

Eight of 74 (11%) articles discussed secondary outcomes.^S17,S34,S35,S59,S80,S83,S99,S100^ Two advised that these should be prespecified with justification for their inclusion (25%).^S83,S100^ Further recommendations for confirmatory testing on secondary endpoints are summarized in Figures 2 and 3.

The “correlation between MOCs” was the most frequently discussed subtheme (32/74 articles [43%]). Of these, four (12.5%) stated that researchers should understand the level of correlation among the MOCs under study to inform robust power calculations, either from prior research or interim analyses.^S2,S23,S64,S90^ Discussion of outcomes correlation under certain MTPs appeared in 29/32 articles (91%),^S1,S2,S4,S6,S16,S28,S34,S35,S40,S44,S53,S56,S57,S59,S63-65,S68,S70-72,S76,S92,S95,S99,S100,S102,S113,S117^ of which six highlighted some MTPs that account for the correlation among MOCs, including multivariate testing methods, bootstrap resampling, and Bayesian methods.^S59,S64,S76,S99,S100,S102^ Composite outcomes were also recommended to account for correlated outcomes in three articles (9%).^S23,S65,S70^ The impact of MOCs correlation on power levels and sample size was discussed in four articles (12.5%).^S57,S64,S71,S84^

Application of MOCs in specific trial and clinical settings was discussed in 31/74 articles (42%), detailed in Supplementary file 6. The most frequently described settings were superiority/noninferiority trials (*N* = 12, 39%)^S9,S38,S44,S45,S56,S72,S76,S80,S81,S84,S87,S110^ and adaptive and group sequential trial designs (*N* = 11, 35%).^S1,S34,S35,S39,S43,S45,S74,S90,S112,S115,S118^

##### 3.4.1.2 Reporting

Guidance on reporting the use of MOCs in RCTs was provided in seven of 74 (9.5%) articles. This included reporting the impact of MOCs and MTPs on type I and II errors in a trial so that readers can reach informed conclusions, ^S19^ following CONSORT checklists for robust reporting of outcomes and changes to prespecified outcomes to avoid data dredging, ^S21,S100^ and reporting unexpected results alongside the rigorously adjusted results to aid generation of new hypotheses.^S5,S68^

One article advised journal editors to evaluate the rationale for the trial and multiplicity adjustment and consider publishing the research even if results are not statistically significant.^S5^

##### 3.4.1.3 Challenges of using MOCs in RCTs

The challenges of using MOCs in trials were described in 23/123 articles (19%). Three subthemes were identified: (1) MOCs in specific research areas (*N* = 11/23, 48%), (2) specific MOCs methods (*N* = 7/23, 30%), and (3) complex trials designs (*N* = 5/23, 22%).

Of the 11 articles discussing specific research areas, three (27%) discussed MOCs in adaptive trials^S34,S35,S74^ whereas challenges across quality of life,^S38^ cost-effectiveness,^S62^ and orthodontics trials^S65^ were each discussed in one article. Areas for further research were also suggested (*N* = 7, 64%) and summarized in Supplementary file 6^S34,S40,S44,S55,S100,S110,S117^.

Several challenges of specific MOCs handling methods were identified across seven articles, detailed in Supplementary file 6. These include the risk of solely relying on unadjusted *P* values and confidence intervals to draw conclusions,^S5^ global testing methods,^S2,S88^ composite,^S51,S70,S110^ and coprimary outcomes^S64^.

On trials complexity, identified challenges included trial efficiency and bias due to individually testing correlated MOCs,^S27,S53^ decisions often being based on labeling of primary or secondary rather than on multiplicity adjustment strategies,^S80^ regional variations in multiplicity adjustment recommendations creating compliance difficulty within multiregional trials,^S78^ and that the control of the experiment-wise or family-wise error rate might not sufficiently reflect the hierarchy of clinical importance in primary outcomes.^S35^ General difficulties including adherence to prespecified elements,^S2^ and impact of low sample size, compliance rates, and effect sizes of MOCs on study power in complex trials were also reported.^S27^

#### 3.4.2 Guidance on the use of specific approaches for handling MOCs

Two specific approaches to multiple primary outcomes were identified in the articles:

##### 3.4.2.1 Coprimary and hierarchical primary outcomes

The use of coprimary and hierarchical outcomes (ie, testing outcomes in a predefined order) was discussed in six of the 74 articles (8%). The two methods’ features and methodological recommendations are summarized in Table 3.^S59,S64,S76,S80,S81,S99^ Additional guidance on similar statistical approaches was provided in 20/74 (27%) articles, further detailed in Supplementary file 6.^S5,S15,S34,S35,S43,S48,S53,S56,S59,S64,S71,S72,S74,S76,S86,S95,S99,S108,S110,S118^

**Table 3:** Summary of the most reported subthemes related to coprimary and hierarchical outcomes

Coprimary outcomes features	Hierarchical outcomes features
Coprimary outcomes should have equal importance for the researcher, and if there is difficulty ascertaining this, input from clinicians and/or patients can be sought.	Hierarchy of outcomes should be based on importance of the endpoints from the clinician and/or researcher perspective.
The power of the coprimary outcomes will be less than the power of each individual outcome. Hence, power and sample size implications are a special challenge in this regard *(known as reverse multiplicity problem)*.	No conclusions should be based on hypotheses failed to be rejected or that falls below these in the hierarchy.

Analyses methods recommendations
Guidance on the following statistical procedures was discussed (see Supplementary file 6 for further resources): - Gatekeeper procedures - Sequential or stepwise testing - Closed testing procedures
Testing secondary endpoints after confirming statistical significance on primary endpoint(s) can follow gatekeeper or closed testing approaches.

##### 3.4.2.2 Composite primary outcomes

Table 4 summarizes the features^S14,S17,S21,S23,S51,S59,S65,S76,S77,S79,S98,S99,S102,S110^ alongside advantages and disadvantages of composite outcomes^S4,S6,S14,S21,S23,S51,S64,S65,S68,S70,S76,S77,S79,S80,S98,S99,S102,S117^ as discussed in 14 and 18 articles, respectively. In addition, recommendations for their analysis,^S6,S23,S51,S59,S64,S68,S76,S77,S80,S99,S102,S104,S107,S110^ reporting,^S21,S51,S59,S68,S77,S80,S99^ and additional challenges are summarized.^S51,S70,S110^

**Table 4:** Summary of the most reported composite outcomes related subthemes identified in the reviewed articles

Features	Advantages	Disadvantages
Composite outcome and its component should be predefined alongside the intended conclusions for the components (confirmatory vs exploratory).	Improves study power and lowers sample size requirements, in turn making trials more feasible.	Difficulty in interpretation resulting from unequal importance of the components and potential variations in treatment impact on the components of a composite.
Components should be biologically related but not highly correlated, clinically meaningful and interpretable, related to the primary objective of the RCT, and be amenable to be impacted by the study treatment.^a^	Resolves the need to control the multiplicity for the primary analysis of the composite.	This can lead to masking treatment impact on composite and the most important component, resulting in the need for a secondary analysis of the components with or without MTPs^b^
Hierarchy of components to be meaningful and reflective of importance for clinicians and patients.	Can produce a comprehensive assessment of the patient experience, hence be of more clinical relevance.	Varying directions of treatment effects on the components may result in loss of study power and inaccurate conclusions.
-	Combining outcomes into a summary measure will have similar benefits to a composite outcome.	-

Analysis methods	Reporting recommendations	Additional challenges
The most frequently reported method of assessment was time-to-event analysis.	***Recommendations for authors:*** reporting the components of the composite outcome as secondary endpoints along with their hierarchical structure.	Using time-to-event analysis can be challenging in terms of deciding what event to account for.
Other methods for composite endpoint analysis discussed are responder-based, Z-score, and rank-based analyses, alongside DOOR, benefit-risk analysis, and win-ratio test.^c^	***Recommendations for readers:*** evaluate the use of the composite, its component, and the time of assessment before arriving to conclusions.	There is a need for more testing procedures for analysis of treatment effect heterogeneity on composite components.
Secondary analysis of the composite components is recommended to assess consistency of treatment effects on components compared to the impact on the composite.	***Recommendations for editors:*** ensure that appropriate statistical methods and accurate wording of results are applied.	Interpretation of treatment effect on composite endpoints, specifically in noninferiority trials, can be especially challenging if treatment impact is mixed at the components level.

a RCT, randomized controlled trial.

b MTPs, multiplicity testing procedures.

c DOOR, desirability of outcome ranking.

The need for further research on using composite outcomes in chronic pain,^S23^ obstetrics,^S77^ and orthodontic trials,^S65^ and how composites can be used to report adverse effects^S98^ were discussed, and further details are provided in Supplementary file 6.

### 3.5 Summary of statistical approaches

Majority of the articles providing general guidance discussed pre-existing MTPs, which are listed in Supplementary file 6. Some described the application of these MTPs within a specific context (eg, in a group sequential design or specific clinical area), while others reviewed or compared various MTPs.^S23,S63,S99^ Bonferroni and its derivative procedures (Holm's, Hochberg's, and Hommel's)^S2,S5,S15–17,S19,S40,S43,S56,S59,S63,S65,S68,S71,S76,S79,S95,S97,S99-101,S110,S117^ and Global testing methods (e.g., O'Brien's tests, Hotelling's T^2^, and Wei-Lachin)^S2,S4,S6,S11,S15-18,S22,S40,S45,S46,S48,S56,S59,S63,S68,S76,S95,S99,S102,S108^ were the most frequently referred to, appearing in 23 (31%) and 22 (30%) of the 74 articles, respectively.

Innovative statistical approaches for handling MOCs were described in 53 of the 123 articles (43%). Of these, four (7.5%) described new statistical procedures^S50,S58,S70,S121^ while 49 (92%) described modifications of pre-existing methods. The modifications either involved the mathematical procedures of the pre-existing test or its application. Supplementary file 7 lists the names of the statistical methods identified and its categorization by type, alongside a summary of the overall impact on type I and II errors, and its ability to account for correlation among MOCs.

## 4 Discussion

This scoping review provides an overview of contemporary methodological and regulatory guidance on the use of MOCs in RCTs. The included articles provided recommendations on the design, analysis, and reporting of trials with MOCs and explored the potential challenges of MOCs in RCTs. The key identified themes of this review were (1) prespecification of outcomes and data analysis planning, (2) need for adjustment for multiplicity, (3) considerations of study power and sample size, (4) testing secondary endpoints, (5) considerations on the correlation between MOCs, and (6) MOCs application in specific research or clinical areas. Most of the identified literature focused on statistical considerations for handling MOCs in regulatory trials context. The identified innovative statistical approaches were mostly modifications of pre-existing methods, while a few proposed new statistical procedures. We found limited guidance on handling secondary outcomes although these are often used in RCTs and formal considerations around its multiplicity are rarely applied [8,24].

We identified a number of regulatory documents from FDA, EMA, ICH, and EUnetHTA providing recommendations on the use of MOCs in RCTs, which focus on trials informing licensing of drugs and medical devices [2,4,16]. Additional important considerations on using MOCs in RCTs were identified from the remaining articles that are either not addressed in regulatory and reporting guidance documents or are elaborated in more detail, such as the following examples. First, transparent rationalization for decisions on multiplicity adjustment is recommended by the articles to facilitate informed conclusions on trials' results by readers and decision-makers. Second, MOCs correlation, power, and sample size considerations, and their implications on MTPs among other challenges were discussed in-depth in these articles. Third, recommendations for reporting RCTs with MOCs were more detailed in the included articles. For instance, there is limited guidance from SPIRIT, CONSORT, and their outcomes extensions on reporting rationale for the secondary outcomes, secondary analyses of composite outcome components, and rationale for MTPs implemented for primary and secondary outcomes; suggestive of some areas where reporting guidance can be improved. The recently published “*WHO Guidance for best practices for clinical trials”* provide recommendations for improving trials practice and robustness of evidence [25]. While it does not specifically address MOCs issues, several of these apply to the context of MOCs, such as the emphasis on selecting appropriate and relevant outcomes, ensuring appropriate sample size, and prespecification of appropriate analysis plans and mitigation procedures that control systematic errors in trials.

Furthermore, our review shows that the guidance on using MOCs in RCTs has typically focused on statistical considerations and is largely limited in the context of nonregulatory settings, including CI trials. Some approaches that increase the complexity of RCTs or the interventions under evaluation are becoming increasingly popular. These include addressing multiple interacting contextual factors and stakeholders in CI development and evaluation [26], reporting on Core Outcome Sets (COS) [27], and implementing flexible yet complex trial designs such as adaptive trials [28,29]. While the benefits of these practices are well-acknowledged, the consequent requirement for MOCs requires guidance on the wider nonstatistical methodological aspects of using MOCs. These include rationalizing the use of MOCs, linking that to the trial's research questions and design. Such rationale should be based on the researcher's thorough understanding of the intervention mechanisms of action—so-called “program theory” [26,30]. This understanding facilitates the process of outcomes selection and may aid in classifying outcomes into primary, secondary, and exploratory. Prespecifying outcomes classification was a key issue identified in our review (Supplementary file 6) and highlights the existing confusion of what stands as a primary, secondary, or exploratory outcome despite of this being a focus of guidance from ICH E9, FDA, and other literature [2,4,[31], [32], [33], [34]]. The issue of these outcomes labels requires careful considerations. A statistically significant primary outcome will lead efficacy claims. Statistically significant secondary endpoint(s) that are only tested after the primary proves significant and for which multiplicity is adjusted can support labeling claims, whereas exploratory endpoints are mostly explanatory (eg, of mechanisms of action) or aim to generate future research hypotheses. Therefore, multiplicity control is not required for exploratory endpoints, and these are mostly not accepted to contribute to efficacy/labeling claims. This also emphasizes that decisions on outcomes classification and addressing its multiplicity (eg, choice of MTP or adjustment approach) need to follow clearly defined research hypotheses and questions/objectives with a consensus among relevant stakeholders (including patients/public involvement) to achieve trials objectives most efficiently. In fact, the lack of clarity and precision of research questions has been identified as a cause of outcomes proliferation [31]. This process can be more complicated and less clear in CIs trials considering the diverse research interests of stakeholders and complexity of interventions and its context.

This study identifies several areas for further research, in addition to research gaps emphasized in the reviewed articles (Supplementary file 6). Using mixed methods approaches to gain key insights from relevant stakeholders can enhance our understanding of the complexities of the underlying statistical and nonstatistical issues of MOCs in RCTs alongside exploring relevant areas for action, particularly for RCTs in the nonregulatory setting. Moreover, since the publication of Vickerstaff's et al review of practices on MOCs in RCTs in 2015 [8], FDA guidance has been updated [4], and recommendations from EMA [16], SPIRIT-Outcomes [17], and CONSORT-Outcomes [18] have been published alongside the publication of another 41 articles identified in our review. Therefore, we will seek to undertake a contemporary reassessment to examine current research practice and explore whether the practice and reporting of MOCs has improved. The scoping review shows that current guidance on MOCs can be limited in a number of key contexts, such as nonstatistical issues, nonregulatory settings, CIs and trial designs, and certain fields of studies. Our review seeks to inform future research to fill these gaps in current methodological guidance.

A key strength of this scoping review is the breadth of literature searches which were not limited by timeframe, clinical/research area, or geographical location of articles. Furthermore, the searches included guidance available on websites of healthcare regulatory agencies, in addition to the bibliographic databases. However, our study has some limitations. We only included articles available online and in English due to the limitation of language expertise among the review team. Reference lists of the included articles were not hand searched, which may have resulted in some relevant literature not being included. We excluded articles that discussed multiple comparisons without a special focus on MOCs. However, these articles were excluded by consensus across our research group, and we contend that articles not specifically discussing MOCs were unlikely to add to the review findings. Finally, this review does not seek to provide guidance on detailed statistical methods for handling MOCs in RCTs, although we do include and categorize the identified statistical methods.

## 5 Conclusion

Current guidance and recommendations on handling MOCs focus on the statistical aspects for RCTs undertaken in the regulatory context to support licensing claims of drugs or medical devices. The findings of this scoping review highlight the need for the development of guidance and further research on the broader implications of MOCs in the design, analysis, and reporting of RCTs, including the clarification of rationale for using MOCs, the handling of secondary outcomes multiplicity, and the application of MOCs in nonregulatory setting, including CIs.

## CRediT authorship contribution statement

**Hadeel Hussein:** Writing – original draft, Visualization, Project administration, Methodology, Formal analysis, Conceptualization. **Rod S. Taylor:** Writing – review & editing, Supervision, Methodology, Formal analysis, Conceptualization. **Anthony Muchai Manyara:** Writing – review & editing, Resources, Methodology, Formal analysis. **Anthony Purvis:** Writing – review & editing, Resources, Methodology. **Richard Emsley:** Writing – review & editing, Supervision. **Rui Duarte:** Writing – review & editing. **Valerie Wells:** Writing – review & editing, Validation, Resources. **Yimin Jiang:** Writing – review & editing, Methodology. **Grace O. Dibben:** Writing – review & editing, Supervision, Formal analysis, Conceptualization.

## Declaration of competing interest

The authors declare that they have no known competing financial interests or personal relationships that could have appeared to influence the work reported in this paper.

## Data Availability

No data was used for the research described in the article.

## Footnotes

1 There is an overlap between these figures as four articles provided both general guidance and statistical method description.
