# Supplementary material for: The need for further guidance on the handling of multiple outcomes in randomized controlled trials: a scoping review of the methodological literature
Source: J Clin Epidemiol. 2025 May;181:None. doi: 10.1016/j.jclinepi.2025.111724 (PMC12289522; doi:10.1016/j.jclinepi.2025.111724)
Supplement: Supplementary files 1–8 [file mmc1.docx]

# Supplementary file 1

Table 1: Preferred Reporting Items for Systematic reviews and Meta-Analyses extension for Scoping Reviews (PRISMA-ScR) Checklist.

| **SECTION** | **ITEM** | **PRISMA-ScR CHECKLIST ITEM** | **REPORTED ON PAGE #** |
| --- | --- | --- | --- |
| **TITLE** | | | |
| Title | 1 | Identify the report as a scoping review. | 1 |
| **ABSTRACT** | | | |
| Structured summary | 2 | Provide a structured summary that includes (as applicable): background, objectives, eligibility criteria, sources of evidence, charting methods, results, and conclusions that relate to the review questions and objectives. | 2 |
| **INTRODUCTION** | | | |
| Rationale | 3 | Describe the rationale for the review in the context of what is already known. Explain why the review questions/objectives lend themselves to a scoping review approach. | 4 |
| Objectives | 4 | Provide an explicit statement of the questions and objectives being addressed with reference to their key elements (e.g., population or participants, concepts, and context) or other relevant key elements used to conceptualize the review questions and/or objectives. | 4-5 |
| **METHODS** | | | |
| Protocol and registration | 5 | Indicate whether a review protocol exists; state if and where it can be accessed (e.g., a Web address); and if available, provide registration information, including the registration number. | 5 |
| Eligibility criteria | 6 | Specify characteristics of the sources of evidence used as eligibility criteria (e.g., years considered, language, and publication status), and provide a rationale. | 5 |
| Information sources* | 7 | Describe all information sources in the search (e.g., databases with dates of coverage and contact with authors to identify additional sources), as well as the date the most recent search was executed. | 5 |
| Search | 8 | Present the full electronic search strategy for at least 1 database, including any limits used, such that it could be repeated. | Supplementary file 2 |
| Selection of sources of evidence† | 9 | State the process for selecting sources of evidence (i.e., screening and eligibility) included in the scoping review. | 5 |
| Data charting process‡ | 10 | Describe the methods of charting data from the included sources of evidence (e.g., calibrated forms or forms that have been tested by the team before their use, and whether data charting was done independently or in duplicate) and any processes for obtaining and confirming data from investigators. | 6 |
| Data items | 11 | List and define all variables for which data were sought and any assumptions and simplifications made. | N/A |
| Critical appraisal of individual sources of evidence§ | 12 | If done, provide a rationale for conducting a critical appraisal of included sources of evidence; describe the methods used and how this information was used in any data synthesis (if appropriate). | N/A |
| Synthesis of results | 13 | Describe the methods of handling and summarizing the data that were charted. | 6 |
| **RESULTS** | | | |
| Selection of sources of evidence | 14 | Give numbers of sources of evidence screened, assessed for eligibility, and included in the review, with reasons for exclusions at each stage, ideally using a flow diagram. | Page 6 and Figure 1 |
| Characteristics of sources of evidence | 15 | For each source of evidence, present characteristics for which data were charted and provide the citations. | Page 7-9 and  Supplementary file 4 |
| Critical appraisal within sources of evidence | 16 | If done, present data on critical appraisal of included sources of evidence (see item 12). | N/A |
| Results of individual sources of evidence | 17 | For each included source of evidence, present the relevant data that were charted that relate to the review questions and objectives. | Supplementary files 5, 6, and 7 |
| Synthesis of results | 18 | Summarize and/or present the charting results as they relate to the review questions and objectives. | Page 6-17 |
| **DISCUSSION** | | | |
| Summary of evidence | 19 | Summarize the main results (including an overview of concepts, themes, and types of evidence available), link to the review questions and objectives, and consider the relevance to key groups. | 6-17 |
| Limitations | 20 | Discuss the limitations of the scoping review process. | 19 |
| Conclusions | 21 | Provide a general interpretation of the results with respect to the review questions and objectives, as well as potential implications and/or next steps. | 17-19 |
| **FUNDING** | | | |
| Funding | 22 | Describe sources of funding for the included sources of evidence, as well as sources of funding for the scoping review. Describe the role of the funders of the scoping review. | Pages 1, 8, 20,  Supplementary file 4 |

# Supplementary file 2

Table 2: Search strategy implemented on each of the bibliographic databases.

| **Database** | **Search strategy** |
| --- | --- |
| **Pubmed** | ("composite score"[Title] OR "multiple outcome*"[Title] OR "multiple comparison*"[Title] OR "composite outcome*"[Title] OR "Multiple endpoints"[Title]) |
| **Embase (OVID)** | ("composite score" or "multiple outcome*" or "multiple comparison*" or "composite outcome*" or "Multiple endpoints").ti. |
| **Science citation Index and Social Science Citation Index (Via Web of Science)** | ("composite score" or "multiple outcome*" or "multiple comparison*" or "composite outcome*" or "Multiple endpoints") (Title) |

# Supplementary file 3

## Reference list of the original search

S1. Baayen C, Hougaard P, Pipper CB. A Versatile Adaptive Dose-Finding Design Based on Multiple Endpoints. *Statistics in Biopharmaceutical Research*. 2017;9(3):302-313. doi:https://dx.doi.org/10.1080/19466315.2017.1341333

S2. Bagiella E. Clinical Trials in Rehabilitation: Single or Multiple Outcomes? *Archives of Physical Medicine and Rehabilitation*. 2009;90(11 SUPPL. 1):S17-S21. doi:https://dx.doi.org/10.1016/j.apmr.2009.08.133

S3. Baldwin SA, Imel ZE, Braithwaite SR, Atkins DC. Analyzing multiple outcomes in clinical research using multivariate multilevel models. *Journal of Consulting and Clinical Psychology*. 2014;82(5):920-930. doi:https://dx.doi.org/10.1037/a0035628

S4. Baraniuk S, Seay R, Sinha AK, Piller LB. Comparison of the global statistical test and composite outcome for secondary analyses of multiple coronary heart disease outcomes. *Progress in Cardiovascular Diseases*. 2012;54(4):357-361. doi:https://dx.doi.org/10.1016/j.pcad.2011.11.001

S5. Barnett MJ, Doroudgar S, Khosraviani V, Ip EJ. Multiple comparisons: To compare or not to compare, that is the question. *Research in social & administrative pharmacy : RSAP*. 2022;18(2):2331-2334. doi:https://dx.doi.org/10.1016/j.sapharm.2021.07.006

S6. Bebu I, Lachin JM. Properties of composite time to first event versus joint marginal analyses of multiple outcomes. *Statistics in Medicine*. 2018;37(27):3918-3930. doi:https://dx.doi.org/10.1002/sim.7849

S7. Bebu I, Lachin JM. Large sample inference for a win ratio analysis of a composite outcome based on prioritized components. *Biostatistics (Oxford, England)*. 2016;17(1):178-187. doi:https://dx.doi.org/10.1093/biostatistics/kxv032

S8. Begun JM, Gabriel KR. CLOSURE OF THE NEWMAN-KEULS MULTIPLE COMPARISONS PROCEDURE. *Journal of the American Statistical Association*. 1981;76(374):241-245. doi:10.2307/2287817

S9. Bloch DA, Lai TL, Su Z, Tubert-Bitter P. A combined superiority and non-inferiority approach to multiple endpoints in clinical trials. *Statistics in Medicine*. 2007;26(6):1193-1207. doi:https://dx.doi.org/10.1002/sim.2611

S10. Bloch DA, Lai TL, Tubert-Bitter P. One-sided tests in clinical trials with multiple endpoints. *Biometrics*. 2001;57(4):1039-1047. doi:http://dx.doi.org/10.1111/j.0006-341X.2001.01039.x

S11. Bregenzer T, Lehmacher W. Directional tests for the analysis of clinical trials with multiple endpoints allowing for incomplete data. *Biometrical Journal*. 1998;40(8):911-928. doi:10.1002/(sici)1521-4036(199812)40:8<911::Aid-bimj911>3.0.Co;2-w

S12. Bretz F, Posch M, Glimm E, Klinglmueller F, Maurer W, Rohmeyer K. Graphical approaches for multiple comparison procedures using weighted Bonferroni, Simes, or parametric tests. *Biometrical Journal*. 2011;53(6):894-913. doi:https://dx.doi.org/10.1002/bimj.201000239

S13. Chang M, Chow SC. Analysis strategies for adaptive designs with multiple endpoints. *Journal of Biopharmaceutical Statistics*. 2007;17(6):1189-1200. doi:https://dx.doi.org/10.1080/10543400701645348

S14. Chang S, Davidson PM, Newton PJ, et al. Composite outcome measures in a pragmatic clinical trial of chronic heart failure management: A comparative assessment. *International Journal of Cardiology*. 2015;185:62-68. doi:https://dx.doi.org/10.1016/j.ijcard.2015.03.071

S15. Chi GYH. Multiple testings: Multiple comparisons and multiple endpoints. *Drug Information Journal*. 1998;32(4 SUPPL.):1347S-1362S. doi:https://dx.doi.org/10.1177/00928615980320s131

S16. Comelli M, Klersy C. Different methods to analyze clinical experiments with multiple endpoints: A comparison on real data. *Journal of Biopharmaceutical Statistics*. 1996;6(2):115-125. doi:http://dx.doi.org/10.1080/10543409608835127

S17. Dueck A, Novotny PJ, Sloan JA. Dealing with Multiple Endpoints. *Current Problems in Cancer*. 2006;30(6):298-306. doi:https://dx.doi.org/10.1016/j.currproblcancer.2006.08.007

S18. Eaton ML, Muirhead RJ. On a multiple endpoints testing problem. *Journal of Statistical Planning and Inference*. 2007;137(11):3416-3429. doi:10.1016/j.jspi.2007.03.021

S19. Feise RJ. Do multiple outcome measures require p-value adjustment? *BMC medical research methodology*. 2002;2:1-4. doi:https://dx.doi.org/10.1186/1471-2288-2-8

S20. Follmann D. Multivariate tests for multiple endpoints in clinical trials. *Stat Med*. 1995;14(11):1163-75. doi:10.1002/sim.4780141103

S21. Freemantle N, Calvert M, Wood J, Eastaugh J, Griffin C. Composite outcomes in randomized trials: greater precision but with greater uncertainty? *JAMA : the journal of the American Medical Association*. 2003;289(19):2554-2559.

S22. Frick H. COMPARING TRIALS WITH MULTIPLE OUTCOMES - THE MULTIVARIATE ONE-SIDED HYPOTHESIS WITH UNKNOWN COVARIANCES. *Biometrical Journal*. 1995;37(8):909-917. doi:10.1002/bimj.4710370803

S23. Gewandter JS, McDermott MP, Evans S, et al. Composite outcomes for pain clinical trials: considerations for design and interpretation. *Pain*. 2021;162(7):1899-1905. doi:https://dx.doi.org/10.1097/j.pain.0000000000002188

S24. Glimm E, Maurer W, Bretz F. Hierarchical testing of multiple endpoints in group-sequential trials. *Statistics in Medicine*. 2010;29(2):219-228. doi:https://dx.doi.org/10.1002/sim.3748

S25. Gong J, Pinheiro JC, Demets DL. Estimating significance level and power comparisons for testing multiple endpoints in clinical trials. *Controlled Clinical Trials*. 2000;21(4):313-329. doi:https://dx.doi.org/10.1016/S0197-2456%2800%2900049-0

S26. Gou J. Sample size optimization and initial allocation of the significance levels in group sequential trials with multiple endpoints. *Biometrical journal Biometrische Zeitschrift*. 2022;64(2):301-311. doi:https://dx.doi.org/10.1002/bimj.202000081

S27. Guo L, Qian Y, Xie H. Assessing complier average causal effects from longitudinal trials with multiple endpoints and treatment noncompliance: An application to a study of Arthritis Health Journal. *Statistics in Medicine*. 2022;41(13):2448-2465. doi:https://dx.doi.org/10.1002/sim.9364

S28. Haneuse S, Schrag D, Nevo D. Invited commentary: Opportunities that come with studying the co-occurrence of multiple outcomes. *American Journal of Epidemiology*. 2020;189(9):982-984. doi:https://dx.doi.org/10.1093/aje/kwaa031

S29. Hasler M. Multiple contrast tests for multiple endpoints in the presence of heteroscedasticity. *International Journal of Biostatistics*. 2014;10(1):17-28. doi:https://dx.doi.org/10.1515/ijb-2012-0015

S30. Hasler M, Hothorn LA. A dunnett-type procedure for multiple endpoints. *International Journal of Biostatistics*. 2011;7(1):3. doi:https://dx.doi.org/10.2202/1557-4679.1258

S31. He T, Liu R, Liu M, Lin J. PMED: Optimal Bayesian Platform Trial Design with Multiple Endpoints. *Journal of Biopharmaceutical Statistics*. 2022;doi:https://dx.doi.org/10.1080/10543406.2022.2080692

S32. Hong H, Chu H, Zhang J, Carlin BP. A Bayesian missing data framework for generalized multiple outcome mixed treatment comparisons. *Research synthesis methods*. 2016;7(1):6-22. doi:https://dx.doi.org/10.1002/jrsm.1153

S33. Huang P, Woolson RF, O'Brien PC. A rank-based sample size method for multiple outcomes in clinical trials. *Statistics in Medicine*. 2008;27(16):3084-3104. doi:https://dx.doi.org/10.1002/sim.3182

S34. Hung HMJ, Wang SJ, O'Neill R. Statistical considerations for testing multiple endpoints in group sequential or adaptive clinical trials. *Journal of Biopharmaceutical Statistics*. 2007;17(6):1201-1210. doi:https://dx.doi.org/10.1080/10543400701645405

S35. Hung HMJ, Wang SJ. Multiple comparisons in complex clinical trial designs. *Biometrical Journal*. 2013;55(3):420-429. doi:https://dx.doi.org/10.1002/bimj.201200048

S36. Huque MF, Alosh M. A flexible fixed-sequence testing method for hierarchically ordered correlated multiple endpoints in clinical trials. *Journal of Statistical Planning and Inference*. 2008;138(2):321-335. doi:10.1016/j.jspi.2007.06.009

S37. James S. Approximate multinormal probabilities applied to correlated multiple endpoints in clinical trials. *Stat Med*. 1991;10(7):1123-35. doi:10.1002/sim.4780100712

S38. Jatoi I, Gail MH. The Need for Combined Assessment of Multiple Outcomes in Noninferiority Trials in Oncology. *JAMA Oncology*. 2020;6(3):420-424. doi:https://dx.doi.org/10.1001/jamaoncol.2019.5361

S39. Jin M, Zhang P. An adaptive seamless Phase 2-3 design with multiple endpoints. *Statistical Methods in Medical Research*. 2021;30(4):1143-1151. doi:https://dx.doi.org/10.1177/0962280220986935

S40. Karrison T, O'Brien P. A rank-sum-type test for paired data with multiple endpoints. *Journal of Applied Statistics*. 2004;31(2):229-238. doi:10.1080/0266476032000148885

S41. Kennedy EH, Kangovi S, Mitra N. Estimating scaled treatment effects with multiple outcomes. *Statistical Methods in Medical Research*. 2019;28(4):1094-1104. doi:https://dx.doi.org/10.1177/0962280217747130

S42. Kieser M, Kirchner M, Dolger E, Gotte H. Optimal planning of phase II/III programs for clinical trials with multiple endpoints. *Pharmaceutical Statistics*. 2018;17(5):437-457. doi:https://dx.doi.org/10.1002/pst.1861

S43. Kieser M, Bauer P, Lehmacher W. Inference on multiple endpoints in clinical trials with adaptive interim analyses. *Biometrical Journal*. 1999;41(3):261-277. doi:10.1002/(sici)1521-4036(199906)41:3<261::Aid-bimj261>3.3.Co;2-l

S44. Kong L, Kohberger RC, Koch GG. Type I error and power in noninferiority/equivalence trials with correlated multiple endpoints: An example from vaccine development trials. *Journal of Biopharmaceutical Statistics*. 2004;14(4):893-907. doi:https://dx.doi.org/10.1081/BIP-200035454

S45. Kropf S, Hommel G, Schmidt U, Brickwedel J, Jepsen MS. Multiple comparisons of treatments with stable multivariate tests in a two-stage adaptive design, including a test for non-inferiority. *Biometrical Journal*. 2000;42(8):951-965. doi:10.1002/1521-4036(200012)42:8<951::Aid-bimj951>3.0.Co;2-#

S46. Lachin JM. Applications of the Wei-Lachin multivariate one-sided test for multiple outcomes on possibly different scales. *PLoS One*. 2014;9(10):e108784. doi:https://dx.doi.org/10.1371/journal.pone.0108784

S47. Lehmacher W, Wassmer G, Reitmeir P. Procedures for two-sample comparisons with multiple endpoints controlling the experimentwise error rate. *Biometrics*. 1991;47(2):511-21.

S48. Lehmacher W, Wassmer G, Reitmeir P. Comment on: on the design and analysis of randomized clinical trials with multiple endpoints. *Biometrics*. 1994;50(2):581-3.

S49. Li QZ, Liu AY, Yu K, Yu KF. A weighted rank-sum procedure for comparing samples with multiple endpoints. *Statistics and Its Interface*. 2009;2(2):197-201.

S50. Li ZB, Cao F, Zhang JJ, Li QZ. Summation of absolute value test for multiple outcome comparison with moderate effect. *Journal of Systems Science & Complexity*. 2013;26(3):462-469. doi:10.1007/s11424-012-0272-5

S51. Lim E, Brown A, Helmy A, Mussa S, Altman DG. Composite outcomes in cardiovascular research: a survey of randomized trials. *Annals of Internal Medicine*. 2008;149(9):612-617.

S52. Lin X, Ryan L, Sammel M, Zhang D, Padungtod C, Xu X. A scaled linear mixed model for multiple outcomes. *Biometrics*. 2000;56(2):593-601. doi:http://dx.doi.org/10.1111/j.0006-341X.2000.00593.x

S53. Liu Y, Hsu J, Ruberg S. Partition testing in dose-response studies with multiple endpoints. *Pharmaceutical Statistics*. 2007;6(3):181-192. doi:https://dx.doi.org/10.1002/pst.295

S54. Logan BR, Tamhane AC. Combining global and marginal tests to compare two treatments on multiple endpoints. *Biometrical Journal*. 2001;43(5):591-604. doi:10.1002/1521-4036(200109)43:5<591::Aid-bimj591>3.0.Co;2-f

S55. Maity A, Williams PL, Ryan L, Missmer SA, Coull BA, Hauser R. Analysis of in vitro fertilization data with multiple outcomes using discrete time-to-event analysis. *Statistics in Medicine*. 2014;33(10):1738-1749. doi:https://dx.doi.org/10.1002/sim.6050

S56. Mascha EJ, Turan A. Joint hypothesis testing and gatekeeping procedures for studies with multiple endpoints. *Anesthesia and Analgesia*. 2012;114(6):1304-1317. doi:https://dx.doi.org/10.1213/ANE.0b013e3182504435

S57. Mishra A, Harichandrakumar KT, Vs B, Satheesh S, Nair NS. Multivariate approach in analyzing medical data with correlated multiple outcomes: An exploration using ACCORD trial data. *Clinical Epidemiology and Global Health*. 2021;11:100785. doi:https://dx.doi.org/10.1016/j.cegh.2021.100785

S58. Montgomery RN, Mahnken JD. A prediction-based test for multiple endpoints. *Statistics in Medicine*. 2020;39(28):4267-4280. doi:https://dx.doi.org/10.1002/sim.8724

S59. Neuhauser M. How to deal with multiple endpoints in clinical trials. *Fundamental and Clinical Pharmacology*. 2006;20(6):515-523. doi:https://dx.doi.org/10.1111/j.1472-8206.2006.00437.x

S60. Nishikawa M, Tango T, Ohtaki M. Statistical tests based on new composite hypotheses in clinical trials reflecting the relative clinical importance of multiple endpoints quantitatively. *Biometrical journal Biometrische Zeitschrift*. 2009;51(5):749-762.

S61. Normand SLT. Multiple outcomes and multiple sources of evidence best statistical practices. *Circulation: Cardiovascular Quality and Outcomes*. 2011;4(6):579-580. doi:https://dx.doi.org/10.1161/CIRCOUTCOMES.111.963751

S62. Nystrand C, Sampaio F, Hoch JS, Osman F, Feldman I. The cost-effectiveness of a culturally tailored parenting program: estimating the value of multiple outcomes. *Cost Effectiveness and Resource Allocation*. 2021;19(1)doi:10.1186/s12962-021-00278-4

S63. O'Brien PC, Geller NL. Interpreting tests for efficacy in clinical trials with multiple endpoints. *Controlled Clinical Trials*. 1997;18(3):222-227. doi:https://dx.doi.org/10.1016/S0197-2456%2897%2900049-4

S64. Offen W, Chuang-Sfein C, Dmitrienko A, et al. Multiple co-primary endpoints: Medical and statistical solutions - A report from the Multiple Endpoints Expert Team of the Pharmaceutical Research and Manufacturers of America. *Drug Information Journal*. 2007;41(1):31-46. doi:http://dx.doi.org/10.1177/009286150704100105

S65. Pandis N. Multiplicity 2: Multiple treatments and multiple outcomes. *American Journal of Orthodontics and Dentofacial Orthopedics*. 2013;143(4):589-591. doi:https://dx.doi.org/10.1016/j.ajodo.2013.01.007

S66. Papageorgiou SN. Planning and interpreting the sample size of trials with multiple outcomes. *Journal of orthodontics*. 2019;46(1):74-76. doi:https://dx.doi.org/10.1177/1465312519831196

S67. Perlman MD, Wu L, Bloch DA, Lai TL, Tubert-Bitter P. A Note on One-Sided Tests with Multiple Endpoints. *Biometrics*. 2004;60(1):276-280. doi:https://dx.doi.org/10.1111/j.0006-341X.2004.00159.x

S68. Pocock SJ. Clinical trials with multiple outcomes: A statistical perspective on their design, analysis, and interpretation. *Controlled Clinical Trials*. 1997;18(6):530-545. doi:https://dx.doi.org/10.1016/S0197-2456%2897%2900008-1

S69. Pocock SJ, Geller NL, Tsiatis AA. The analysis of multiple endpoints in clinical trials. *Biometrics*. 1987;43(3):487-98.

S70. Pogue J, Devereaux PJ, Thabane L, Yusuf S. Designing and analyzing clinical trials with composite outcomes: Consideration of possible treatment differences between the individual outcomes. *PLoS One*. 2012;7(4):e34785. doi:https://dx.doi.org/10.1371/journal.pone.0034785

S71. Porter KE. Statistical Power in Evaluations That Investigate Effects on Multiple Outcomes: A Guide for Researchers. *Journal of Research on Educational Effectiveness*. 2018;11(2):267-295. doi:10.1080/19345747.2017.1342887

S72. Quan H, Bolognese J, Yuan W. Assessment of equivalence on multiple endpoints. *Statistics in Medicine*. 2001;20(21):3159-3173. doi:https://dx.doi.org/10.1002/sim.985

S73. Quan H, Luo X, Capizzi T. Multiplicity adjustment for multiple endpoints in clinical trials with multiple doses of an active treatment. *Statistics in Medicine*. 2005;24(14):2151-2170. doi:https://dx.doi.org/10.1002/sim.2101

S74. Rauch G, Kieser M. Adaptive designs for clinical trials with multiple endpoints. *Clinical Investigation*. 2015;5(5):433-435. doi:https://dx.doi.org/10.4155/cli.14.138

S75. Reitmeir P, Wassmer G. Resampling-based methods for the analysis of multiple endpoints in clinical trials. *Statistics in Medicine*. 1999;18(24):3453-3462. doi:https://dx.doi.org/10.1002/%28SICI%291097-0258%2819991230%2918:24%3C3453::AID-SIM283%3E3.0.CO;2-Z

S76. Ristl R, Urach S, Rosenkranz G, Posch M. Methods for the analysis of multiple endpoints in small populations: A review. *Journal of Biopharmaceutical Statistics*. 2019;29(1):1-29. doi:https://dx.doi.org/10.1080/10543406.2018.1489402

S77. Ross S. Composite outcomes in randomized clinical trials: arguments for and against. *American Journal of Obstetrics and Gynecology*. 2007;196(2):e1-119. doi:https://dx.doi.org/10.1016/j.ajog.2006.10.903

S78. Sakamaki K, Yoshida S, Morita Y, et al. Challenges on Multiple Endpoints in Clinical Trials: An Industry Survey in Japan. *Therapeutic Innovation and Regulatory Science*. 2020;54(3):528-533. doi:https://dx.doi.org/10.1007/s43441-019-00084-4

S79. Schouten HJA. Combined evidence from multiple outcomes in a clinical trial. *Journal of Clinical Epidemiology*. 2000;53(11):1137-1144. doi:https://dx.doi.org/10.1016/S0895-4356%2800%2900238-9

S80. Snapinn S. Some remaining challenges regarding multiple endpoints in clinical trials. *Statistics in Medicine*. 2017;36(28):4441-4445. doi:https://dx.doi.org/10.1002/sim.7390

S81. Song JX. Sample size for simultaneous testing of rate differences in non-inferiority trials with multiple endpoints. *Computational Statistics & Data Analysis*. 2009;53(4):1201-1207. doi:10.1016/j.csda.2008.10.028

S82. Stallard N, Thall PF, Whitehead J. Decision theoretic designs for phase II clinical trials with multiple outcomes. *Biometrics*. 1999;55(3):971-977. doi:http://dx.doi.org/10.1111/j.0006-341X.1999.00971.x

S83. Stone A, Chuang-Stein C. Strong control over multiple endpoints: Are we adding value to the assessment of medicines? *Pharmaceutical Statistics*. 2013;12(4):189-191. doi:https://dx.doi.org/10.1002/pst.1574

S84. Sun A, Dong X, Tsong Y. Sample size determination for equivalence assessment with multiple endpoints. *Journal of Biopharmaceutical Statistics*. 2014;24(6):1203-1214. doi:https://dx.doi.org/10.1080/10543406.2014.941986

S85. Sun H, Kawaguchi A, Koch G. Analyzing multiple endpoints in a confirmatory randomized clinical trial-an approach that addresses stratification, missing values, baseline imbalance and multiplicity for strictly ordinal outcomes. *Pharmaceutical Statistics*. 2017;16(2):157-166. doi:https://dx.doi.org/10.1002/pst.1799

S86. Sun H, Snyder E, Koch GG. Statistical planning in confirmatory clinical trials with multiple treatment groups, multiple visits, and multiple endpoints. *Journal of Biopharmaceutical Statistics*. 2018;28(1):189-211. doi:https://dx.doi.org/10.1080/10543406.2017.1378664

S87. Tamhane MC, Logan BR. A superiority-equivalence approach to one-sided tests on multiple endpoints in clinical trials. *Biometrika*. 2004;91(3):715-727.

S88. Tang DI, Geller NL. Closed testing procedures for group sequential clinical trials with multiple endpoints. *Biometrics*. 1999;55(4):1188-1192. doi:http://dx.doi.org/10.1111/j.0006-341X.1999.01188.x

S89. Tang DI, Geller NL, Pocock SJ. On the design and analysis of randomized clinical trials with multiple endpoints. *Biometrics*. 1993;49(1):23-30.

S90. Tang DI, Gnecco C, Geller NL. DESIGN OF GROUP SEQUENTIAL CLINICAL-TRIALS WITH MULTIPLE ENDPOINTS. *Journal of the American Statistical Association*. 1989;84(407):776-779. doi:10.2307/2289665

S91. Teixeira-Pinto A, Mauri L. Statistical analysis of noncommensurate multiple outcomes. *Circulation: Cardiovascular Quality and Outcomes*. 2011;4(6):650-656. doi:https://dx.doi.org/10.1161/CIRCOUTCOMES.111.961581

S92. Teixeira-Pinto A, Siddique J, Gibbons R, Normand SL. Statistical Approaches to Modeling Multiple Outcomes in Psychiatric Studies. *Psychiatric Annals*. 2009;39(7):729-735. doi:10.3928/00485713-20090625-08

S93. Thall PF, Sung HG. Some extensions and applications of a Bayesian strategy for monitoring multiple outcomes in clinical trials. *Statistics in Medicine*. 1998;17(14):1563-1580. doi:https://dx.doi.org/10.1002/%28SICI%291097-0258%2819980730%2917:14%3C1563::AID-SIM873%3E3.0.CO;2-L

S94. Tilley BC, Pillemer SR, Heyse SP, Li S, Clegg DO, Alarcón GS. Global statistical tests for comparing multiple outcomes in rheumatoid arthritis trials. MIRA Trial Group. *Arthritis Rheum*. 1999;42(9):1879-88. doi:10.1002/1529-0131(199909)42:9<1879::Aid-anr12>3.0.Co;2-1

S95. Tilley BC, Marler J, Geller NL, et al. Use of a global test for multiple outcomes in stroke trials with application to the National Institute of Neurological Disorders and Stroke t- PA Stroke Trial. *Stroke*. 1996;27(11):2136-2142. doi:http://dx.doi.org/10.1161/01.STR.27.11.2136

S96. Troendle JF, Legler JM. A comparison of one-sided methods to identify significant individual outcomes in a multiple outcome setting: Stepwise tests or global tests with closed testing. *Statistics in Medicine*. 1998;17(11):1245-1260. doi:https://dx.doi.org/10.1002/%28SICI%291097-0258%2819980615%2917:11%3C1245::AID-SIM833%3E3.0.CO;2-Z

S97. Troendle JF. A permutational step-up method of testing multiple outcomes. *Biometrics*. 1996;52(3):846-859. doi:http://dx.doi.org/10.2307/2533047

S98. Tugwell P, Judd MG, Fries JF, Singh G, Wells GA. Powering our way to the elusive side effect: A composite outcome 'basket' of predefined designated endpoints in each organ system should be included in all controlled trials. *Journal of Clinical Epidemiology*. 2005;58(8):785-790. doi:https://dx.doi.org/10.1016/j.jclinepi.2004.11.028

S99. Turk DC, Dworkin RH, McDermott MP, et al. Analyzing multiple endpoints in clinical trials of pain treatments: IMMPACT recommendations. *Pain*. 2008;139(3):485-493. doi:https://dx.doi.org/10.1016/j.pain.2008.06.025

S100. Tyler KM, Normand SLT, Horton NJ. The use and abuse of multiple outcomes in randomized controlled depression trials. *Contemporary Clinical Trials*. 2011;32(2):299-304. doi:https://dx.doi.org/10.1016/j.cct.2010.12.007

S101. Vickerstaff V, Omar RZ, Ambler G. Methods to adjust for multiple comparisons in the analysis and sample size calculation of randomised controlled trials with multiple primary outcomes. *BMC medical research methodology*. 2019;19(1):129. doi:https://dx.doi.org/10.1186/s12874-019-0754-4

S102. Vickerstaff V, Ambler G, Omar RZ. A comparison of methods for analysing multiple outcome measures in randomised controlled trials using a simulation study. *Biometrical journal Biometrische Zeitschrift*. 2021;63(3):599-615. doi:https://dx.doi.org/10.1002/bimj.201900040

S103. Wang B, Cui X. A new partition testing strategy for multiple endpoints. *Statistics in Medicine*. 2012;31(20):2151-2168. doi:https://dx.doi.org/10.1002/sim.5366

S104. Wang H, Peng J, Zheng JZ, et al. Win Ratio -An Intuitive and Easy-To-Interpret Composite Outcome in Medical Studies. *Shanghai Arch Psychiatry*. 2017;29(1):55-60. doi:10.11919/j.issn.1002-0829.217011

S105. Wang L, Chen Y, Zhu HJ. Implementing optimal allocation in clinical trials with multiple endpoints. *Journal of Statistical Planning and Inference*. 2017;182:88-99. doi:10.1016/j.jspi.2016.09.002

S106. Wang SJ. A closed procedure based on Follmann's test for the analysis of multiple endpoints. *Communications in Statistics-Theory and Methods*. 1998;27(10):2461-2480. doi:10.1080/03610929808832237

S107. Wang Z, Chen J. Testing for Trend in Benefit-Risk Analysis with Prioritized Multiple Outcomes. *Statistics in Biopharmaceutical Research*. 2020;12(3):380-389. doi:https://dx.doi.org/10.1080/19466315.2019.1690037

S108. Wassmer G, Reitmeir P, Kieser M, Lehmacher W. Procedures for testing multiple endpoints in clinical trials: An overview. *Journal of Statistical Planning and Inference*. 1999;82(1-2):69-81. doi:10.1016/s0378-3758(99)00032-4

S109. Westfall PH, Ho SY, Prillaman BA. Properties of multiple intersection-union tests for multiple endpoints in combination therapy trials. *J Biopharm Stat*. 2001;11(3):125-38. doi:10.1081/bip-100107653

S110. Wiens BL. Multiple comparisons in non-inferiority trials: Reaction to recent regulatory guidance on multiple endpoints in clinical trials. *Journal of Biopharmaceutical Statistics*. 2018;28(1):52-62. doi:https://dx.doi.org/10.1080/10543406.2017.1378667

S111. Wiens BL. A fixed sequence Bonferroni procedure for testing multiple endpoints. *Pharmaceutical Statistics*. 2003;2(3):211-215. doi:10.1002/pst.64

S112. Xi D, Tamhane AC. Allocating recycled significance levels in group sequential procedures for multiple endpoints. *Biometrical journal Biometrische Zeitschrift*. 2015;57(1):90-107. doi:https://dx.doi.org/10.1002/bimj.201300157

S113. Xiong C, Yu K, Gao F, Yan Y, Zhang Z. Power and sample size for clinical trials when efficacy is required in multiple endpoints: Application to an Alzheimer's treatment trial. *Clinical Trials*. 2005;2(5):387-393. doi:https://dx.doi.org/10.1191/1740774505cn112oa

S114. Xu H, Nuamah I, Liu J, Lim P, Sampson A. A Dunnett-Bonferroni-based parallel gatekeeping procedure for dose-response clinical trials with multiple endpoints. *Pharmaceutical Statistics*. 2009;8(4):301-316. doi:https://dx.doi.org/10.1002/pst.358

S115. Xu T, Qin Q, Wang X. Defining information fractions in group sequential clinical trials with multiple endpoints. *Contemporary Clinical Trials Communications*. 2018;10:77-79. doi:https://dx.doi.org/10.1016/j.conctc.2018.03.005

S116. Xu X, Pennell ML, Lu B, Murray DM. Efficient Bayesian joint models for group randomized trials with multiple observation times and multiple outcomes. *Statistics in Medicine*. 2012;31(24):2858-2871. doi:https://dx.doi.org/10.1002/sim.5414

S117. Yoon FB, Fitzmaurice GM, Lipsitz SR, Horton NJ, Laird NM, Normand SLT. Alternative methods for testing treatment effects on the basis of multiple outcomes: Simulation and case study. *Statistics in Medicine*. 2011;30(16):1917-1932. doi:https://dx.doi.org/10.1002/sim.4262

S118. Zhang FQ, Gou JT. Refined critical boundary with enhanced statistical power for non-directional two-sided tests in group sequential designs with multiple endpoints. *Statistical Papers*. 2021;62(3):1265-1290. doi:10.1007/s00362-019-01134-7

S119. Zhang J, Quan H, Ng J, Stepanavage ME. Some statistical methods for multiple endpoints in clinical trials. *Controlled Clinical Trials*. 1997;18(3):204-221. doi:https://dx.doi.org/10.1016/S0197-2456%2896%2900129-8

S120. Zhang P, Liu P, Ma J, Shentu Y. Value Function Guided Subgroup Identification via Gradient Tree Boosting: A Framework to Handle Multiple Outcomes for Optimal Treatment Recommendation. *Statistics in Biopharmaceutical Research*. 2021;doi:https://dx.doi.org/10.1080/19466315.2021.1972832

S121. Zhang W, Liu A, Tang LL, Li Q. A cluster-adjusted rank-based test for a clinical trial concerning multiple endpoints with application to dietary intervention assessment. *Biometrics*. 2019;75(3):821-830. doi:https://dx.doi.org/10.1111/biom.13029

S122. Zhang Z, Wang C, Troendle JF. Optimizing the order of hypotheses in serial testing of multiple endpoints in clinical trials. *Statistics in Medicine*. 2015;34(9):1467-1482. doi:https://dx.doi.org/10.1002/sim.6425

S123. Zhang ZW. REVERSE REGRESSION: A METHOD FOR JOINT ANALYSIS OF MULTIPLE ENDPOINTS IN RANDOMIZED CLINICAL TRIALS. *Statistica Sinica*. 2014;24(4):1753-1769. doi:10.5705/ss.2010.111

S124. Food and Drug Administration (FDA). *Multiple Endpoints in Clinical Trials Guidance for Industry*. 2022.

S125. The International Council for Harmonisation of Technical Requirements for Pharmaceuticals for Human Use (ICH). *<ich-e-9-statistical-principles-clinical-trials-step-5_en.pdf>*. 1998.

S126. European Medicines Agency (EMA). *Guideline on multiplicity issues in clinical trials*. 2016. https://www.ema.europa.eu/en/documents/scientific-guideline/draft-guideline-multiplicity-issues-clinical-trials_en.pdf

S127. European Network for Health Technology Assessment (EUnetHTA). *D4.5 – APPLICABILITY OF EVIDENCE – PRACTICAL GUIDELINE ON MULTIPLICITY, SUBGROUP, SENSITIVITY AND POST HOC ANALYSES*. 2022. https://www.eunethta.eu/wp-content/uploads/2022/12/EUnetHTA21-D4.5-Practical-Guideline-on-Applicability-of-Evidence-v1.0.pdf

S128. An-Wen C, Jennifer MT, Peter CG, et al. SPIRIT 2013 explanation and elaboration: guidance for protocols of clinical trials. *BMJ : British Medical Journal*. 2013;346:e7586. doi:10.1136/bmj.e7586

S129. Schulz KF, Altman DG, Moher D, Group C. CONSORT 2010 Statement: updated guidelines for reporting parallel group randomised trials. *BMC Med*. Mar 24 2010;8:18. doi:10.1186/1741-7015-8-18

S130. Butcher NJ, Monsour A, Mew EJ, et al. Guidelines for Reporting Outcomes in Trial Protocols: The SPIRIT-Outcomes 2022 Extension. *JAMA*. 2022;328(23):2345-2356. doi:10.1001/jama.2022.21243

S131. Butcher NJ, Monsour A, Mew EJ, et al. Guidelines for Reporting Outcomes in Trial Reports: The CONSORT-Outcomes 2022 Extension. *JAMA*. 2022;328(22):2252-2264. doi:10.1001/jama.2022.21022

# Supplementary file 4

Table 3: Detailed characteristics of the articles reviewed from the original database search (N=123).

| **1st Author** | **Publication Year** | **1st author country** | **Affiliation** | **Publication type** | **Description of 'Other Publication type** | **Research area** | **Description of 'Other' clinical area** | **Funding** |
| --- | --- | --- | --- | --- | --- | --- | --- | --- |
| **Baayen, C. ^S1^** | 2017 | France | Consultancy | Research publication | - | Specific research area | Adaptive Dose finding trials | Regulatory agency |
| **Bagiella, E. ^S2^** | 2009 | US | University | Research publication | **-** | Specific research area | Rehabilitation medicine | Funding not reported |
| **Baldwin, S. A. ^S3^** | 2014 | US | University | Research publication | **-** | Specific research area | Psychotherapy research | Public research funding |
| **Baraniuka, S. ^S4^** | 2012 | US | University | Research publication | **-** | Specific research area | Coronary heart disease research | Funding not reported |
| **Barnett, M. J. ^S5^** | 2022 | US | University | Review | - | General guidance | **-** | Funding not reported |
| **Bebu, I. ^S6^** | 2018 | US | University | Research publication | **-** | General guidance | **-** | Other |
| **Bebu, I. ^S7^** | 2016 | US | University | Research publication | **-** | General statistical method | **-** | Public research funding |
| **Begun, J. ^S8^** | 1981 | US | University | Research publication | **-** | General statistical method | **-** | Funding not reported |
| **Bloch, D. A. ^S9^** | 2007 | US | University | Research publication | **-** | Specific research area | Superiority/non-inferiority trials | Public research funding |
| **Bloch, D. ^S10^** | 2001 | US | University | Research publication | **-** | General statistical method | **-** | Public research funding |
| **Bregenzer, T. ^S11^** | 1998 | Germany | University | Research publication | **-** | General guidance | **-** | Funding not reported |
| **Bretz, F. ^S12^** | 2011 | Switzerland | Pharmaceutical company | Research publication | **-** | General statistical method | **-** | Public research funding |
| **Chang, M. ^S13^** | 2007 | US | Pharmaceutical company | Research publication | **-** | Specific research area | Adaptive designs | Funding not reported |
| **Chang, S. ^S14^** | 2015 | Australia | University | Research publication | **-** | Specific research area | Cardiovascular research | Mixed funding sources |
| **Chi, G. ^S15^** | 1998 | US | Regulatory agency | Research publication | **-** | General guidance | **-** | Funding not reported |
| **Comelli, M. ^S16^** | 1996 | Italy | University | Research publication | **-** | General guidance | **-** | Funding not reported |
| **Dueck, A. ^S17^** | 2006 | US | Academic Hospital | Research publication | - | Specific research area | Quality of Life trials | Funding not reported |
| **Eaton, M. L. ^S18^** | 2007 | US | University | Research publication | **-** | General guidance | **-** | Funding not reported |
| **Feise, R. J. ^S19^** | 2002 | US | Consultancy | Other | Debate | General guidance | **-** | Funding not reported |
| **Follmann, D. ^S20^** | 1995 | US | Governmental research agency | Research publication | **-** | General statistical method | **-** | Funding not reported |
| **Freemantle, N. ^S21^** | 2003 | UK | University | Research publication | **-** | General guidance | **-** | Mixed funding sources |
| **Frick, H. ^S22^** | 1995 | Germany | Research institute | Research publication | **-** | General guidance | **-** | Funding not reported |
| **Gewandtera, J. ^S23^** | 2021 | US | University | Research publication | **-** | Specific research area | Pain management | Mixed funding sources |
| **Glimm, E. ^S24^** | 2010 | Switzerland | Pharmaceutical company | Research publication | **-** | Specific research area | Group sequential design | Funding not reported |
| **Gong, J. ^S25^** | 2000 | US | University | Research publication | **-** | General statistical method | **-** | Funding not reported |
| **Gou, J. ^S26^** | 2022 | US | University | Research publication | **-** | Specific research area | Group sequential design | Funding not reported |
| **Guo, L. ^S27^** | 2022 | Canada | University | Research publication | **-** | General statistical method | **-** | Mixed funding sources |
| **Haneuse, S. ^S28^** | 2020 | US | University | Commentary | - | General guidance | **-** | Public research funding |
| **Hasler, M. ^S29^** | 2014 | Germany | University | Research publication | **-** | General statistical method | **-** | Funding not reported |
| **Hasler, M. ^S30^** | 2011 | Germany | University | Research publication | **-** | General statistical method | **-** | Funding not reported |
| **He, T. ^S31^** | 2022 | US | University | Research publication | **-** | General statistical method | **-** | No funding received |
| **Hong, H. ^S32^** | 2016 | US | University | Research publication | **-** | General statistical method | **-** | Funding not reported |
| **Huang, P. ^S33^** | 2008 | US | University | Research publication | **-** | General guidance | **-** | Public research funding |
| **Hung, H. M. J. ^S34^** | 2007 | US | Regulatory agency | Research publication | **-** | Specific research area | Adaptive/Group sequential design | Regulatory agency |
| **Hung, H. M. J. ^S35^** | 2013 | US | Regulatory agency | Research publication | **-** | Specific research area | Complex trials design | Funding not reported |
| **Huque, M. F. ^S36^** | 2008 | US | Regulatory agency | Research publication | **-** | General statistical method | **-** | Funding not reported |
| **James, S. ^S37^** | 1991 | UK | University | Research publication | **-** | General statistical method | **-** | Funding not reported |
| **Jatoi, I. ^S38^** | 2020 | US | University | Research publication | **-** | Specific research area | Non-inferiority trials in oncology | Public research funding |
| **Jin, M. ^S39^** | 2021 | US | Pharmaceutical company | Research publication | **-** | Specific research area | Adaptive designs phase II/III trials | Pharmaceutical company |
| **Karrison, T. ^S40^** | 2004 | US | University | Research publication | - | Guidance and statistics | **-** | University or Research institute |
| **Kennedy, E. H. ^S41^** | 2019 | US | University | Research publication | **-** | General statistical method | **-** | Other |
| **Kieser, M. ^S42^** | 2018 | Germany | University | Research publication | **-** | Specific research area | Phase II/III trials | Public research funding |
| **Kieser, M. ^S43^** | 1999 | Germany | Pharmaceutical company | Research publication | **-** | Specific research area | Adaptive interim analysis design/ Group sequential design | Funding not reported |
| **Kong, L. ^S44^** | 2004 | US | University | Research publication | **-** | Specific research area | Non-inferiority/equivalence trials | Funding not reported |
| **Kropf, S. ^S45^** | 2000 | Germany | University | Research publication | **-** | Specific research area | Adaptive design, non-inferiority | Funding not reported |
| **Lachin, J. M. ^S46^** | 2014 | US | University | Research publication | **-** | General guidance | **-** | Public research funding |
| **Lehmacher, W. ^S47^** | 1991 | Germany | Research institute | Research publication | **-** | General statistical method | **-** | Funding not reported |
| **Lehmacher, W. ^S48^** | 1994 | Germany | Research institute | Other | Correspondence letter | Guidance and statistics | **-** | Funding not reported |
| **Li, Q. ^S49^** | 2009 | US | governmental research agency | Research publication | **-** | General statistical method | **-** | Public research funding |
| **Li, Z. ^S50^** | 2013 | China | University | Research publication | **-** | General statistical method | **-** | Public research funding |
| **Lim, E. ^S51^** | 2008 | UK | Academic Hospital | Research publication | - | Specific research area | Cardiovascular research | No conflict declared/No funding received |
| **Lin, X. ^S52^** | 2000 | US | University | Research publication | **-** | General statistical method | **-** | Public research funding |
| **Liu, Y. ^S53^** | 2007 | US | University | Research publication | **-** | Specific research area | Dose finding trials | Public research funding |
| **Logan, B. R. ^S54^** | 2001 | US | University | Research publication | **-** | General statistical method | **-** | Funding not reported |
| **Maity, A. ^S55^** | 2014 | US | University | Research publication | **-** | Specific research area | In vitro fertilisation (reproductive health research) | Public research funding |
| **Mascha, E. J. ^S56^** | 2012 | US | Academic Hospital | Research publication | **-** | Specific research area | Superiority/non-inferiority trials | University or Research institute |
| **Mishra, A. ^S57^** | 2021 | India | University | Research publication | **-** | General guidance | **-** | Funding not reported |
| **Montgomery, R. N. ^S58^** | 2020 | US | University | Research publication | **-** | General statistical method | **-** | Public research funding |
| **Neuhauser, M. ^S59^** | 2006 | Germany | University | Review | **-** | General guidance | **-** | Funding not reported |
| **Nishikawa, M. ^S60^** | 2009 | Japan | governmental research agency | Research publication | **-** | General statistical method | **-** | Funding not reported |
| **Normand, S. L. T. ^S61^** | 2011 | US | University | Other | Editor's perspective | Specific research area | Cardiovascular research | Mixed funding sources |
| **Nystrand, C. ^S62^** | 2021 | Sweden | University | Research publication | **-** | Specific research area | Cost-effectiveness | Mixed funding sources |
| **O’Brien, P. C. ^S63^** | 1997 | US | Academic Hospital | Research publication | - | General guidance | **-** | Funding not reported |
| **Offen, W. ^S64^** | 2007 | US | Pharmaceutical company | Other | Report | General guidance | **-** | Pharmaceutical company |
| **Pandis, N. ^S65^** | 2013 | Switzerland & Greece | Journal associate editor | Editorial | - | Specific research area | Orthodontics research | Funding not reported |
| **Papageorgiou, S. N. ^S66^** | 2019 | Switzerland | University | Research publication | - | General guidance | **-** | Funding not reported |
| **Perlman, M. D. ^S67^** | 2004 | US | University | Other | A note | General statistical method | **-** | Funding not reported |
| **Pocock, S. J. ^S68^** | 1997 | UK | University | Research publication | **-** | General guidance | **-** | Funding not reported |
| **Pocock, S. J. ^S69^** | 1987 | UK | University | Research publication | **-** | General statistical method | **-** | Other |
| **Pogue, J. ^S70^** | 2012 | Canada | University | Research publication | - | Guidance and statistics | **-** | Mixed funding sources |
| **Porter, K. E. ^S71^** | 2018 | US | Research institute | Research publication | **-** | General guidance | **-** | Public research funding |
| **Quan, H. ^S72^** | 2001 | US | Pharmaceutical company | Research publication | **-** | Specific research area | Equivalence trials | Funding not reported |
| **Quan, H. ^S73^** | 2005 | US | Pharmaceutical company | Research publication | **-** | Specific research area | Multiple doses trials | Funding not reported |
| **Rauch, G. ^S74^** | 2015 | Germany | University | Editorial | - | Specific research area | Adaptive design | Other |
| **Reitmeir, P. ^S75^** | 1999 | Germany | governmental research agency | Research publication | **-** | General statistical method | **-** | Funding not reported |
| **Ristl, R. ^S76^** | 2019 | Austria | University | Research publication | **-** | Specific research area | Small population setting | Regulatory agency |
| **Ross, S. ^S77^** | 2006 | Canada | University | Other | Clinical opinion | Specific research area | Composite outcomes in obstetric trials | Funding not reported |
| **Sakamaki, K. ^S78^** | 2020 | Japan | University | Research publication | **-** | General guidance | **-** | University or Research institute |
| **Schouten, H. J. A. ^S79^** | 2000 | Netherlands | University | Research publication | **-** | General guidance | **-** | Funding not reported |
| **Snapinn, S. ^S80^** | 2017 | US | Biotechnology company | Other | Featured Article | General guidance | **-** | Funding not reported |
| **Song, J. X. ^S81^** | 2009 | US | Pharmaceutical company | Research publication | **-** | Specific research area | Non-inferiority trials | Funding not reported |
| **Stallard, N. ^S82^** | 1999 | UK | University | Research publication | **-** | Specific research area | Phase II trials | Mixed funding sources |
| **Stone, A. ^S83^** | 2013 | UK | Pharmaceutical company | Other | Viewpoint article | General guidance | **-** | Funding not reported |
| **Sun, A. ^S84^** | 2014 | US | Regulatory agency | Research publication | **-** | Specific research area | Equivalence trials | Funding not reported |
| **Sun, H. ^S85^** | 2017 | US | University | Research publication | **-** | Specific research area | Confirmatory trials | Funding not reported |
| **Sun, H. ^S86^** | 2018 | US | University | Research publication | - | Guidance and statistics | - | Funding not reported |
| **Tamhane, M. C. ^S87^** | 2004 | US | University | Research publication | **-** | Specific research area | Superiority-equivalence trials | Funding not reported |
| **Tang, D. I. ^S88^** | 1999 | US | Research institute | Research publication | **-** | Specific research area | Group sequential design | Public research funding |
| **Tang, D. I. ^S89^** | 1993 | US | Research institute | Research publication | **-** | General statistical method | **-** | Public research funding |
| **Tang, D. I. ^S90^** | 1989 | US | Research institute | Research publication | **-** | Specific research area | Group sequential design | Public research funding |
| **Teixeira-Pinto, A. ^S91^** | 2011 | Portugal | University | Research publication | **-** | General statistical method | **-** | Public research funding |
| **Teixeira-Pinto, A. ^S92^** | 2009 | Portugal | University | Research publication | **-** | Specific research area | Psychiatry research | Public research funding |
| **Thall, P. F. ^S93^** | 1998 | US | University | Research publication | **-** | General statistical method | **-** | Funding not reported |
| **Tilley, B. C. ^S94^** | 1999 | US | University | Research publication | **-** | Specific research area | Rheumatoid arthritis trials | Public research funding |
| **Tilley, B. C. ^S95^** | 1996 | US | Academic Hospital | Other | Workshop summary | Specific research area | Neurology research | Public research funding |
| **Troendle, J. F. ^S96^** | 1998 | US | governmental research agency | Research publication | **-** | General statistical method | **-** | Public research funding |
| **Troendle, J. F. ^S97^** | 1996 | US | Governmental research agency | Research publication | **-** | General guidance | **-** | Funding not reported |
| **Tugwell, P. ^S98^** | 2005 | Canada | University | Research publication | **-** | Specific research area | Adverse events | Funding not reported |
| **Turk, D. C. ^S99^** | 2008 | US | University | Research publication | **-** | Specific research area | Pain management | Pharmaceutical company |
| **Tyler, K. M. ^S100^** | 2011 | US | University | Research publication | **-** | Specific research area | Depression trials | Mixed funding sources |
| **Vickerstaff, V. ^S101^** | 2019 | UK | University | Research publication | **-** | General guidance | **-** | Mixed funding sources |
| **Vickerstaff, V. ^S102^** | 2021 | UK | University | Research publication | - | General guidance | **-** | Public research funding |
| **Wang, B. ^S103^** | 2012 | US | Pharmaceutical company | Research publication | **-** | General statistical method | **-** | Funding not reported |
| **Wang, H. ^S104^** | 2017 | US | University | Research publication | **-** | General guidance | **-** | Funding not reported |
| **Wang, L. ^S105^** | 2017 | US | University | Research publication | **-** | General guidance | **-** | Mixed funding sources |
| **Wang, S. J. ^S106^** | 1998 | US | Regulatory agency | Research publication | **-** | General statistical method | **-** | Funding not reported |
| **Wang, Z. ^S107^** | 2020 | US | University | Research publication | **-** | Specific research area | Benefit-risk analyses | Funding not reported |
| **Wassmer, G. ^S108^** | 1999 | Germany | University | Research publication | **-** | General guidance | **-** | Funding not reported |
| **Westfall, P. H. ^S109^** | 2001 | US | University | Research publication | **-** | Specific research area | Combination therapy trials | Funding not reported |
| **Wiens, B. L. ^S110^** | 2018 | US | Pharmaceutical company | Research publication | **-** | Specific research area | Non-inferiority trials | Funding not reported |
| **Wiens, B. L. ^S111^** | 2003 | US | Pharmaceutical company | Research publication | **-** | General statistical method | **-** | Funding not reported |
| **Xi, D. ^S112^** | 2015 | US | Pharmaceutical company | Research publication | **-** | Specific research area | Group sequential design | Funding not reported |
| **Xiong, C. ^S113^** | 2005 | US | University | Research publication | **-** | General guidance | **-** | Public research funding |
| **Xu, H. ^S114^** | 2009 | US | Pharmaceutical company | Research publication | **-** | Specific research area | Dose response trials | Funding not reported |
| **Xu, T. ^S115^** | 2018 | US | Pharmaceutical company | Other | Manuscript | Specific research area | Group sequential design | Pharmaceutical company |
| **Xu, X. ^S116^** | 2012 | US | University | Research publication | **-** | Specific research area | Group randomised trials | Public research funding |
| **Yoon, F. B. ^S117^** | 2011 | US | University | Research publication | **-** | General guidance | **-** | Public research funding |
| **Zhang, F. ^S118^** | 2021 | US | University | Research publication | **-** | Specific research area | Group sequential design | Funding not reported |
| **Zhang, J. ^S119^** | 1997 | US | Pharmaceutical company | Research publication | **-** | General statistical method | **-** | Funding not reported |
| **Zhang, P. ^S120^** | 2021 | US | Pharmaceutical company | Research publication | **-** | General statistical method | **-** | Funding not reported |
| **Zhang, W. ^S121^** | 2019 | US | governmental research agency | Research publication | **-** | General statistical method | **-** | Public research funding |
| **Zhang, Z. ^S122^** | 2015 | US | Regulatory agency | Research publication | **-** | General statistical method | **-** | Funding not reported |
| **Zhang, Z. ^S123^** | 2014 | US | Regulatory agency | Research publication | **-** | General statistical method | **-** | Public research funding |

# Supplementary file 5

Table 4: Summary of the FDA 2022 guidance for industry on handling multiple outcomes.^S124^

| **Subheading** | **Main guidance points** |
| --- | --- |
| ***MULTIPLE ENDPOINTS: GENERAL PRINCIPLES*** | |
| **The hierarchy of families of endpoints** | Grouped hierarchy is based on clinical significance, expected frequency of occurrence of the endpoint, and anticipated effects. In addition, a key factor is whether the relevant endpoint is intended to establish treatment effect or reveal additional meaningful effects.   - *Primary Endpoint Family:*   Endpoint(s) that establish treatment effect(s) to conclude whether the trial meets its objectives. Can be used in three ways:  (a) a positive effect on any of the primary endpoints is sufficient for drug efficacy ≫ multiplicity adjustment is required.  (b) all primary endpoints are required to show positive effect for drug efficacy (co-primary endpoints) ≫ no multiplicity adjustment is required.  (c) primary endpoints are combined in a composite or a multi-component endpoint to avoid multiplicity issues.   - *Secondary and Exploratory Endpoint Families:*   Can be used to demonstrate evidence on further clinical impact(s) related to or distinct to the primary endpoint. Multiplicity adjustment needs to be considered and methods pre-specified in planning stage. Using higher number of secondary endpoints lowers the likelihood of detecting an impact of any of them, hence limiting the number of secondary endpoints is preferable. If a treatment effect is detected on the primary endpoint, positive secondary endpoints can then be interpretable. Multiplicity adjustment should account for Type I error rate for primary as well as secondary outcomes. Where a clinically important event is expected to have earlier/more frequent occurrence than another clinically important event that has fewer occurrence, researchers can consider the former to be the primary endpoint and the latter be used as secondary endpoint. This will ensure larger study power.  Multiplicity adjustment is not needed for exploratory endpoints. |
| **Type II error rate and sample size** | Study power is defined as the likelihood that a treatment effect of a particular size is present, and it determines the success of the study. The sample size is designed to have an appropriately high power to detect a treatment effect on the primary endpoint if in fact such an effect exists. Hence, the study power is a crucial factor for choosing the sample size, particularly for the primary outcomes. Considerations of the statistical adjustment of multiplicity to control Type I error rate should be included in calculating the sample size. The Type II error rate increases when co-primary endpoints are used, hence study power is reduced. Calculations of sample size in such case will depend on whether correlation between endpoints exists and, on the power required for each endpoint. |
| **Types of multiple endpoints** | 1. ***When Demonstration of Treatment Effects on Two or More Distinct Endpoints Is Recommended to Establish Clinical Benefit (Co-Primary Endpoints):***  - Multiplicity correction is not required. - Type II error is raised with the use of co-primary endpoints. Thus, the use of more than 2 co-primary outcomes should be carefully examined to limit the loss of power, unless highly clinically significant. - Raising alpha level for each of the co-primary outcomes should not be practiced as this will impact the interpretability of treatment effect on each outcome, hence undermine the treatment approval.  1. ***When Demonstration of a Treatment Effect on at Least One of Several Primary Endpoints Is Sufficient:***  - This method can be used when it is unknown a priori which endpoint/disease aspect will be affected by the treatment under study. - Here, a treatment effect on any of several endpoints can be acceptable to conclude treatment effectiveness, creating a primary endpoint *family.* - Multiplicity adjustment to control type I error is necessary.  1. ***Composite Endpoints:***  - Defined as ‘the occurrence or realization in a subject of any one of the specified components.’ - There is no multiplicity issue when one statistical test is conducted for the composite outcome. - It is crucial that the components of a composite outcome are analysed as this can impact the interpretation of trial results and will allow more detailed knowledge treatment effect. whether to adjust for multiplicity or not will be based on the purpose of these analyses; no adjustment needed if it is intended to support understanding of impact on composite endpoint, however, adjustment is required if the purpose is to detect other benefits of the drug that will be described in the FDA-approved labelling. - Composite outcomes allow the use of a reasonable sample size and duration of study for a suitable study power as it provides higher event rate compared to individual event components.  1. ***Multi-Component Endpoints:***  - Defined as ‘a within-subject combination of two or more components’. - Specified components are observed and assessed for each subject individually, followed by a single overall rating, score, or status that is based on predetermined rules. - The overall score can be an average or a dichotomous outcome. Pre-specification of what is considered as positive response or a required degree of improvement on each component can be done. - Although this is an efficient type of endpoint when there is a similar trend of treatment impact on the components, study power can be undermined if such harmony among the components is limited. - Clinical ruling is used to decide on appropriate components to include, rather than statistical considerations.  1. ***Clinically Critical Endpoints Too Infrequent for Use as a Primary Endpoint:***  - In such case, the infrequent endpoint can be part of a composite outcome. - A positive impact on the composite outcome should be followed by a secondary analysis (pre-planned) to aid with conclusion of treatment impact on that component. |
| **The individual components of composite and other multi-component endpoints** | ***Evaluating and Reporting the Results of Composite Endpoints***   - Composite components that correspond to events are defined ‘as the first occurrence’ of that event. - Proportions of events can be used to compare study groups at the conclusion of the study. Other means of analysing these events is via time-to-event analysis, especially when the event-free time has clinical considerations. - When separate hypotheses are intended to analyse of particular composite endpoint component(s), these should be included in the predefined plan of statistical analysis and multiplicity adjustment. These analysis tests, however, can be underpowered as the sample size/total event rate are determined based on the composite endpoint. - When decomposition of the first composite is used, analysis plan should account for all events for each event type with appropriate multiplicity adjustment. This is because events after the first events would not be counted with the decomposition of first composite event and such analyses may demonstrate further interpretable treatment effects.   ***Evaluating and Reporting the Results on Other Multi-Component Endpoints***   - As with composite endpoints, analysis of individual components of multi-component endpoints is important and can be done if clinically interpretable. Appropriate multiplicity correction is to be prespecified and applied upon analysis. |
| ***STATISTICAL METHODS*** | |
| **Statistical methods illustrated by the FDA 2022 guidance report** | Bonferroni method |
|  | Holm procedure |
|  | Hochberg procedure |
|  | Prospective Alpha Allocation Scheme |
|  | The Fixed-Sequence Method |
|  | Resampling-Based, Multiple-Testing Procedures |
|  | Gatekeeping Testing Strategies |
|  | Graphical Approaches Based on Sequentially Rejective Tests |
| ***Other regulatory and reporting guidelines were identified from*** | |
| ICH (the International Council for Harmonisation of Technical Requirements for Pharmaceuticals for Human Use), ^S125^ EMA (European Medicines Agency), ^S126^ EUnetHTA (European Network for Health Technology Assessment), ^S127^ SPIRIT (Standard Protocol Items: Recommendations for Interventional Trials) and CONSORT (Consolidated standards for Reporting of Trials) core statements, ^S128,S129^ and Outcomes extensions. ^S130,S131^ | |

# Supplementary file 6

Table 5: Detailed themes and subthemes on general and specific approaches to MOCs synthesised from 74 articles with mapping of the items against FDA 2022 guidance *(similarities with FDA are in bold)*.^[[1]](#footnote-1)^

| **Main theme** | **Subtheme** | **Summary of recommendations** | **No. of articles** | **Addressed by FDA** |
| --- | --- | --- | --- | --- |
| **Design** | **Pre-specification:** It is essential that a clear plan of handling multiple outcomes is determined a priori and reported in protocols where relevant. It is important that researchers adhere to pre-specified plans and are expected to report and justify any deviation from that plan. ^S100^ The pre-specification should cover the following: | Medical/research questions and hypotheses. These provide the basis for **selection of primary endpoints,** as well as guide the choice of appropriate statistical procedures of MOCs analysis. ^S1,S2,S15,S17,S40,S63,S71,S86,S99,S100^ | 10 | Y (partially) |
|  |  | **Selection of outcomes along with clear specification of the nature of each outcome; primary, secondary, or exploratory.** Authors should consider the need to provide definitions to clarify the nature of each endpoint as this assist in resolving confusion around need for multiplicity adjustment and in interpretation of results. If using a small sample size, pre-specification becomes more crucial. ^S14,S15,S17,S19,S65,S68,S78,S80,S83,S86,S99^ | 11 | Y (partially) |
|  |  | **Clinical decision rule and the outcomes that need to show statistical significance to conclude the desired treatment effect.** This should ideally be based on the clinical importance and relevance for the investigator(s). ^S15,S43,S71,S78,S99,S101^ | 6 | Y (partially) |
|  |  | **The need for multiplicity adjustment and the procedures or approaches to be used along with clear analysis plan.** Authors should clarify the rationale for multiplicity adjustment to help reader and/or decision makers weigh advantages and disadvantages of their approach to multiplicity adjustment.^S5,S19,S68,S71,S78,S86,S99-101^ | 9 | Y (partially) |
|  |  | **Where exploratory endpoints are identified, it is important that these are clearly labelled as such. Results based on exploratory endpoints cannot support claims, however, these can examine or generate future hypotheses.**^S59,S99^ | 2 | Y |
|  | **Significant level adjustment** | **This is needed to strongly control type I error rate (false positives).** ^S2,S5,S19,S34,S65,S99,S100^ Appropriate use of endpoints terminology is essential to avoid confusion over the need for multiplicity adjustment as is the need for an agreement among regulators regarding used terminology.^S78,S80^ Consider the rationale for the clinical decision under study (e.g. regulatory decision making or marketing claim) to guide the decision of whether multiplicity adjustment is required. ^S5,S78^  1 article discussed situations where decision on multiplicity adjustment is challenging. ^S80^ | 9 | Y (partially) |
|  |  | ***When adjustment is needed:***   1. **When statistical significance is required on one or more of several primary endpoints, but not all of them, to conclude positive result.** ^S15,S79,S83,S84,S99,S101^ 2. **Adjustment for multiplicity among secondary endpoints is required where conclusions based on these endpoints are to be included in confirmatory analysis or support label claims.** ^S5,S15,S76,S110^ | 9 | Y |
|  |  | ***When adjustment is not needed:***   1. **When a single primary endpoint is used, and all other endpoints are declared to only have an exploratory role. Such endpoints can be employed to explore mechanisms of action, generate new knowledge for further research, or test secondary hypotheses.** ^S59,S83,S99^ 2. **When statistical significance is required on all primary endpoints to conclude positive results (co-primary outcomes)**. ^S56,S59,S64,S76,S80,S84,S99^ 3. **Safety endpoints: on evaluation of safety endpoints, it is possible to not adjust for multiplicity to avoid false negative results. However, authors may still consider multiplicity correction if there are concerns of type I error inflation.** An alternative is to consider controlling the False Discovery Rate (FDR) (See below). ^S99^ 4. Adjustment is not required with certain approaches of handling multiple outcomes. These include:  - **testing endpoints following a pre-specified hierarchical structure,** ^S34,S59,S76^ - **primary analysis of a composite endpoint.** ^S21,S80,S102,S117^ | 12 | Y (partially) |
|  |  | *False discovery rate:*  As multiplicity adjustment methods will result in an increase of type II error (proportionate to the number of outcomes), controlling the False Discovery Rate might be a potential solution. This approach keeps the false positives rate at a desired level rather than adjusting significance level. ^S71^ Consider controlling the FDR in an exploratory study, when dealing with multiple safety endpoints, in small sample settings, and when using correlated outcomes.^S59,S76,S99^ | 4 | N |
|  | **Secondary endpoints** | **Should be prespecified** with justification for their inclusion.^S83,S100^ | 2 | Y (partially) |
|  |  | The decision of which secondary endpoints to be included in product labelling should be based on experts’ judgment. ^S83^ | 1 | N |
|  |  | **Secondary endpoints that are intended to be included in confirmatory analyses and/or the label of a product must be clearly specified for this purpose in advance and should only be tested after primary endpoint(s) demonstrate statistical significance.** ^S34,S35,S59,S80^ | 4 | Y |
|  |  | If statistical significance is not detected on the primary outcome, it may be still useful to test secondary outcomes. If researcher(s) determined the need to test secondary endpoints, appropriate statistical method for alpha adjustment should be considered. ^S17,S99^ | 2 | N |
|  | **Implications on study power and sample size:**  Adjusting for multiplicity potentially increases type II error due to loss of study power.^S5,S19,S59,S76^ This is more pronounced with higher number of primary endpoints and will depend on correlation between them ^S2,S71,S99^. Hence, adjusting sample size is recommended to ensure adequate study power. Implications include: | **Determine the smallest effect size that would be clinically relevant.** ^S23,S77^ | 2 | Y |
|  |  | **Use as large sample size as possible to avoid underpowered results and issues with bias in reporting or interpretation.** ^S5,S66^ | 2 | Y |
|  |  | Consider the use of a single primary endpoint, a composite endpoint, or a combined assessment method, if appropriate. This resolves the impact of MOCs on study power and sample size by resolving the need to adjust for multiplicity. ^S17,S19,S21,S64,S65,S68,S71,S76,S98^ | 9 | N |
|  |  | Consider the use of a single primary endpoint with multiple secondary endpoints where necessary, e.g. when effect sizes are varied across outcomes. ^S19,S33,S101^ | 3 | N |
|  |  | Consider employing secondary endpoints for sample size and power calculation as this will enhance the study power for the primary endpoint. ^S80^ | 1 | N |
|  |  | Consider the use of a statistical method that can enhance statistical power or have minimal negative impact on power. **This will depend on the number, type, and structure of endpoints as well as the correlation among these and the study purpose.** ^S34,S40,S56,S64,S71,S76,S101^ The impacts of specific multiplicity procedures on power are discussed by several papers.^S1,S2,S5,S33,S35,S38,S48,S65,S68,S70,S79,S80,S99,S102^ Clinical and statistical requirements of the trial and feasibility concerns also impact sample size calculation. ^S19,S66,S76^ | 23 | Y (partially) |
|  | **Correlation** | Caution needed when choosing primary and secondary endpoints to avoid the use of many correlated outcomes as this leads to complex analysis and difficult interpretation. ^S65^ | 1 | N |
|  |  | For robust power calculation, researchers should understand degree of correlation among the outcomes they intend to use. ^S2,S90^ This can be generated from prior research or an interim assessment if appropriate. ^S23,S64^ | 4 | N |
|  |  | Some of the methods that account for correlations include joint hypotheses testing, **bootstrap and** **resampling**, Simes methods, Bayesian methods, and some Multivariate procedures.^S59,S64,S76,S99,S100,S102^ Other articles discussed **correlation under various MTPs**.^[[2]](#footnote-2) S1,S2,S4,S6,S16,S28,S34,S35,S40,S44,S53,S56,S57,S63-65,S68,S70-72,S76,S92,S95,S102,S113,S117^ | 29 | Y (partially) |
|  |  | Composite or combined endpoints can be employed to include correlated components. ^S23,S65,S70^ One article focused on orthodontics trials. ^S65^ This correlation should be accounted for by appropriate statistical methods. ^S70^ | 3 | N |
|  |  | The impact of correlation on power levels and/or sample size in the setting of specific statistical approaches is discussed.^S57,S64,S71,S84^ | 4 | N |
|  | **Specific uses of MOCs:** the use of MOCs in the following specific contexts or fields of study is discussed in some articles. | Designing product brochure and product labels. ^S78,S83,S95^ | 3 | N/A |
|  |  | Implementation and challenges of MOCs in adaptive trial design with recommendations for further research. ^S1,S34,S39,S43,S45,S74^ Group sequential trial design with MOCs is also discussed. ^S34,S35,S43,S90,S112,S115,S118^ | 11 |  |
|  |  | Design, analysis and reporting of superiority/non-inferiority/ equivalence trials in the context of multiple outcomes. ^S9,S38,S44,S45,S56,S72,S76,S80,S81,S84,S87,S110^ 1 article focused on cancer trials setting. ^S38^ | 12 |  |
|  |  | Phase II/III trials. ^S39,S42,S64^ | 3 |  |
|  |  | Quality of life trials. ^S17^ | 1 |  |
|  |  | Multiple endpoints in cost-effectiveness trials. ^S62^ | 1 |  |
|  |  | Using MOCs in the setting of a small sample. ^S11,S76^ | 2 |  |
|  |  | Trial-in-trial design and alpha strategies for trials with MACE outcome^S35^ and the use of composite and global testing strategy in CHD trials.^S4^^[[3]](#footnote-3)^ | 2 |  |
|  |  | Application of Response-adaptive Randomisation (RAR) design in the context of MOCs. ^S105^ | 1 |  |
| **Analysis** | - | **Bonferroni and its derivative methods**^S2,S5,S15-17,S19,S40,S43,S56,S59,S63,S65,S68,S71,S76,S79,S95,S97,S99-101,S110,S117^ | 23 | Y |
|  |  | **Resampling and bootstrap methods**^S5,S9,S15,S16,S59,S76,S97,S99,S108,S110^ | 10 | Y |
|  |  | Multivariate Methods^S5,S76,S92,S102^ | 4 | N |
|  |  | Bayesian approaches^S5,S61,S64^ | 3 | N |
|  |  | James’s test^S16,S59^ | 2 | N |
|  |  | Logistic regression^S16^ | 1 | N |
|  |  | Pocock method^S16^ | 1 | N |
|  |  | Alpha strategies (propagation, spending, **prospective allocation**, recycling, and splitting) ^S35,S39,S99,S110,S112,S115^ | 6 | Y (partially) |
|  |  | Global methods^S2,S4,S6,S11,S15-18,S22,S40,S45,S46,S48,S56,S59,S63,S68,S76,S95,S99,S102,S108^ | 22 | N |
|  |  | Intersection union test (IUT)^S18,S44,S56,S59,S64,S72,S87,S113^ | 8 | N |
|  |  | Partitioning method ^S53,S76^ | 2 | N |
|  |  | Tests for post-hoc comparisons^S5^ | 1 | N |
|  |  | Min test^S44^ | 1 | N |
| **Reporting** | - | Authors should report the level and impact of type I and II errors in their research for the reader to make informed conclusions. ^S19^ | 1 | N |
|  |  | Consider using CONSORT checklist to ensure appropriate reporting of primary and secondary endpoints as well as reporting changes in pre-specified outcomes, hence limit potential data dredging.^[[4]](#footnote-4)S21,S100^ | 2 | N |
|  |  | Adjustment for multiplicity can be restrictive. Therefore**, it is of value to report unexpected or interesting statistical and/or clinical findings while maintaining the more rigorously adjusted results to allow the generation of new hypotheses.** ^S5,S68^ | 2 | Y (partially) |
|  |  | **Reporting results of trials that use rare endpoints as the most important endpoint(s) is crucial**. ^S78^ | 1 | Y |
|  |  | In addition to assessing the quality of statistical approach, statistical significance and the need for adjustment, readers are encouraged to pay attention to the overall quality of the research and the effect sizes. Readers should base their conclusions on all analyses done in a trial and consider the overall evidence base available from other trials. ^S17,S19^ | 2 | N |
|  |  | Editors must evaluate the rationale of the trial, assess the need for multiplicity adjustment and consider the results for publication even if not achieving statistical significance. Requesting the p-values to be reported with fuller explanations can assist in avoiding data dredging. ^S5^ | 1 | N |
| **Challenges** | **Trials complexity** | Problems associated with increased trial’s complexity including low sample sizes, compliance rates, and effect sizes of each of the MOCs lead to low study power. ^S27^ | 1 | N |
|  |  | Testing correlated MOCs separately can lead to lower efficiency, biased results and is an important issue on MOCs research. ^S27,S53^ | 2 | N |
|  |  | - The control of the experiment-wise or family-wise error rate might not sufficiently reflect the clinical hierarchy of importance in primary outcomes, which may warrant further considerations. ^S35^  - Decision and interpretation of results from testing primary, secondary, and/or key secondary endpoints can often be confusingly based on labels rather than multiplicity adjustment performed. ^S80^  - It can be a challenge to set multiplicity adjustment plan for multiregional trials. This is due to variations in definitions and recommendations of multiplicity adjustment procedures. ^S78^ | 3 | N |
|  |  | Challenges that come with using multiple outcomes approach include defining the global hypothesis, determining the best order for hypothesis testing, and deciding which should be part of sample size calculations.^S2^ | 1 | N |
|  | **Challenges on specific MOCs methods** | Rejecting the global hypothesis under Global testing does not necessarily translate into accepting the new treatment. ^S88^ Careful selection of individual outcomes is required to limit challenges of interpretation with prior knowledge or estimation of correlation among outcomes. ^S2^ | 2 | N |
|  |  | Researchers should not rely solely on p-values in case of multiple outcomes as these can be misleading with no multiplicity adjustment. Since confidence intervals are dependent on p-values, these should also be dealt with cautiously. ^S5^ | 1 | N |
|  | **Challenges of MOCs in specific research areas** | Challenges in reporting adverse effects of treatments in trials with regulatory context due to potentially long list of adverse events to report. ^S68^ | 1 | N |
|  |  | In adaptive or group sequential designs, it is statistically more complex to test secondary outcomes only after primary outcomes achieves significance as it becomes harder to achieve the required statistical properties. ^S34,S35^ Further challenges of MOCs in adaptive designs are discussed. ^S74^ | 3 | N |
|  |  | Outcomes related to quality of life can be challenging to measure. ^S38^ | 1 | N |
|  |  | Challenges in cost-effectiveness studies, specifically using clinical outcomes to test cost-effectiveness. ^S62^ | 1 | N |
|  |  | Challenges of MOCs in orthodontics research. ^S65^ | 1 | N |
|  |  | Further research needed in:   - Vaccine studies, ^S44^ - In-vitro fertilisation studies, ^S55^ - CTP in flexible designs (e. g. adaptive and GSD)^[[5]](#footnote-5)^, ^S34^ - Using rank-sum test for binary and ordinal data, ^S40^ - Handling missing data, ^S40,S100^ - Joint testing for non-commensurate outcomes, ^S117^ and - Using novel statistical procedures for MOCs in regulatory settings should be with caution as the results based on such methods can be doubtful for decision makers. Further research should further explore strengths and pitfalls of new methods. ^S110^ | 7 | N |
| **Guidance on specific MOCs approaches** | | | | |
| **Co-primary and hierarchical outcomes** | **Features** | Co-primary endpoints: these should be equally important to the investigator and the rational for using co-primary endpoints should be pre-specified. Clinical and/or patient input can guide the choice of co-primary endpoints when there is difficulty judging the equality of relevance of those endpoints. **The power of co-primary endpoints is lower than the power of each individual outcome. As this disproportionately impact study power with increasing co-primary endpoints,** it can be problematic for small sized trials. ^S64,S76,S81^ | 3 | Y (partially) |
|  |  | **Reverse multiplicity problem.** ^S64,S99^ | 2 | Y |
|  |  | **Gatekeeper and hierarchical methods: Hierarchy of testing is pre-defined and can be based on importance of a hypothesis from clinician or researcher perspective. No conclusions can be based on the hypothesis that failed to be rejected or hypotheses that follows it in hierarchy.** ^S59,S80^ | 2 | Y |
|  | **Specific applications** | The use of co-primary outcomes in Phase II/III is discussed. ^S42,S64^ | 2 | N/A |
|  |  | No adjustment is to be done if sponsors add co-primary endpoints additional to those determined by regulators. However, gatekeeper approach can be followed to test those endpoints for the purpose of inclusion in product label. ^S64^ | 1 |  |
|  | **Analysis** | **Gatekeeping procedures** **can be serial, parallel, or tree structured.** **Serial gatekeeper techniques do not require adjustment for multiplicity** whereas the parallel does. ^S56,S59,S99^ Gatekeeping method is also discussed by other authors. ^S5,S64,S76,S86,S110^ | 8 | Y (partially) |
|  |  | Closed testing procedures. ^S15,S34,S43,S48,S53,S56,S59,S76,S86,S99,S108^ | 11 | N |
|  |  | **Stepwise procedures: can be arranged in a step-up or step-down design as hypotheses are ordered by their importance level. Examples are Hochberg’s test, Holm’s test, and the gatekeeper procedures.** ^S5,S95^ **Those sequential Bonferroni-driven methods can be graphically represented**. ^S76,S110^ Stepwise methods were also discussed in other articles. ^S53,S71,S72,S118^ | 8 | Y |
|  |  | **Testing secondary outcomes for confirmatory purposes can be achieved** by employing gatekeeper, closed testing, or **hierarchical methods.** ^S34,S35,S56,S59,S64,S74,S76^ | 7 | Y (partially) |
|  | **Challenges** | Implications of using co-primary endpoints can be less understood. ^S64^ | 1 | N |
| **Composite outcomes** | **Features** | **Composite endpoint and its components should be pre-specified. ^S14,S21,S59,S77,S79,S102^ Authors should adhere to their pre-specified definitions.** ^S77^ | 6 | Y |
|  |  | Components should be biologically related but not highly correlated, be related to the primary objective of a trial, be of clinical significance and are interpretable. ^S21,S51,S76,S77,S98,S99^ | 6 | N |
|  |  | **The intended conclusions on the components should be pre-specified and multiplicity adjusted appropriately.** ^S79,S99^ | 2 | Y |
|  |  | Rational for choosing the composite, its components and the timing of evaluation need to be pre-specified, and definitions adhered to throughout the trial. ^S77^ | 1 | N |
|  |  | Components and their hierarchy should be meaningful to patients as to clinicians. These should be based on clinicians’ and patients’ judgement as well as prior research. ^S23,S51,S77,S79^ | 4 | N |
|  |  | **Researchers should include components that are predicted to be impacted by the treatment under study.** ^S110^ | 1 | Y |
|  |  | **Importance of each component should be considered so that the change seen in the composite should not be the result of change on the least important component.** ^S14,S51,S77,S110^ Weighing components can enhance validity of the composite, however, it can complicate its interpretation. ^S77^ | 4 | Y (partially) |
|  |  | **Combining endpoint measures into a single statistic or creating a summary measure is another method to approach multiple outcomes similar to composite endpoints.** ^S17,S59,S65,S76,S79^ The less number of components are included in such a measure, the more feasible it will be to implement. ^S79^ | 5 | Y(partially) |
|  |  | It is important to recognize the minimum clinically meaningful difference in advance of the trial to support sample size assessment and interpretation. ^S23,S77,S79^ | 3 | N |
|  |  | Pre-specification should also cover the decision of who will assess the combined outcome (patients and/or researchers). ^S79^ | 1 | N |
|  | **Advantages** | **Improve statistical power. This in turn is reflected on sample size requirements and leads to improved feasibility and resource allocation.** ^S4,S6,S14,S21,S64,S65,S68,S70,S76,S77,S79,S98,S99^ | 13 | Y |
|  |  | **Resolve the need to adjust for multiplicity**. ^S14,S21,S64,S80,S99,S102,S117^ | 7 | Y |
|  |  | **Since it is composed of multiple components, a composite endpoint can be more comprehensive in assessment of patients’ experiences and be of more relevance for investigators or decision makers.** ^S6,S14,S21,S23,S77,S99^ | 6 | Y |
|  |  | Although composite outcomes do not reflect correlation among its components, ^S4^ it can be designed to include information on correlation between components, hence is of particular use for benefit/risk assessment as these components are usually correlated. These can be employed for a benefit/risk profile assessment for individual patients. ^S23,S65,S70^ | 4 | N |
|  | **Disadvantages** | **Components may not have equal importance and may have differing treatment impact. This can lead to challenging interpretation, masking of true impact on the composite or the clinically more important component, and the need for a secondary analysis with or without multiplicity adjustment**. ^S6,S14,S21,S68,S98,S99^ | 6 | Y |
|  |  | Therefore, the use of composite outcomes can lead to poor reporting and/or misinterpretation. ^S21,S23,S51,S77^ | 4 | N |
|  |  | Defining the minimum clinically important difference is complex. ^S77^ | 1 | N |
|  |  | Including death as a component can create difficulty as no further events can be detected for the participant. Further analyses of components aid interpretation of treatment impact on components. ^S51^ | 1 | N |
|  |  | Inclusion of components predicted to be impacted by treatment is usually based on clinician judgment, hence relevance of a composite endpoint to patients may be questionable. ^S21,S77^ | 2 | N |
|  |  | **The common but less important components can have more contribution in a composite endpoint compared to the rarer but more important components**. ^S51,S68^ | 2 | Y |
|  |  | **When treatment effects on components are in different directions or of varying sizes, this can lead to loss of power and/or inaccurate conclusion about the impact on the composite endpoint.** ^S79,S102^ | 2 | Y |
|  | **Specific applications** | Using composite endpoints in the setting of non-inferiority trials. ^S110^ | 1 | N/A |
|  |  | Regulatory bodies assess trials that use composite endpoint individually depending on the composite used and the evidence-base supporting it. ^S110^ | 1 |  |
|  |  | Composite endpoints should not be the basis for marketing claims. ^S21^ | 1 |  |
|  |  | There is a need for a methodological guidance on developing composite outcomes for obstetrics as well as chronic pain research. ^S23,S77^ In obstetrics, a special focus on neonatal long-term composite outcomes is needed. | 2 |  |
|  |  | The use of composite endpoints in orthodontal trials. ^S65^ | 1 |  |
|  |  | Useful evidence can be generated on rare outcomes as components for potential composite endpoints via systematic reviews and meta-analyses. ^S21^ | 1 |  |
|  |  | An article described composite heterogeneity test to assess treatment heterogeneity at the stages of trial design and analysis. ^S70^ | 1 |  |
|  |  | The basket: using composite endpoints to report adverse effects of interventions according to the body organ affected. ^S98^ | 1 |  |
|  | **Analysis** | Researchers should refer to empirical evidence when deciding on assessment method for the composite endpoint occurrence. ^S77^ | 1 | N |
|  |  | **Secondary analysis of composite components is required to assess that the treatment impact on each of the components is consistent with the composite outcome**. ^S23,S59,S80,S99,S110^ | 5 | Y |
|  |  | **Composite endpoints are usually analysed by time-to-event.** ^S6,S51,S64,S68,S76,S80,S102^ Composite endpoints with efficacy and safety domains can be analysed using responder-based analysis or benefit/risk assessment.^S23^ Both methods can be designed in various ways depending on trial objectives. One article compared methods for benefit-risk assessment on composite endpoints. ^S107^ Other suggested methods include Z-Score and Rank-based analysis. ^S23^ | 9 | Y (partially) |
|  |  | Composite endpoints can also be individualized by patients; an example method is the Desirability of Outcome Ranking (DOOR). ^S23^ | 1 | N |
|  |  | Win ratio test is discussed including its interpretation and advantages. ^S6,S104^ | 2 | N |
|  | **Reporting** | Readers should evaluate the use of composite, its components, and time of measurement in a trial before relying on reported conclusions. ^S51,S77,S80^ | 3 | N |
|  |  | **Authors should consider reporting the components of a primary composite endpoint as secondary endpoints along with the hierarchy of their structure.** Thus, readers will have the opportunity to judge its importance to their field. ^S21,S51,S59,S68,S77,S80,S99^ The number of participants experiencing each of the components should also be reported to aid interpretation. ^S51^ | 7 | Y (partially) |
|  |  | Editors must **ensure the appropriate use of statistical methods and appropriate wording of reporting results of a composite endpoint**. ^S21,S77,S80^ | 3 | Y |
|  | **Challenges** | Challenges of composite endpoints:   - Further research is needed on testing treatment heterogeneity between composite components. ^S70^ - **Using time-to-event analysis in composite outcomes where multiple events occur in a participant result in a challenge of deciding which event to account for**. ^S51^ - **Using composite endpoint in non-inferiority trials can have its own challenges on results interpretation where treatment impact is mixed on individual components**. ^S110^ | 3 | Y (partially) |

.

# Supplementary file 7

Table 6: Identified innovative statistical methods (categorised) with summary of impact on type I and II errors and relevance to correlation.^[[6]](#footnote-6)^

| **Category** | **Names of approaches reviewed** | **Impact on Type I error** | **Impact on Type II error** | **Accounting for correlation** |
| --- | --- | --- | --- | --- |
| **Alpha-adjusting methods** | Method based on Bonferroni procedure with estimation of correlation coefficient (for testing the primary and secondary endpoints) ^S25^ | **Alpha allocation/spending/recycling:**  Can control type I error rate. Allocation can be used to assess secondary outcomes after assessment of primary outcomes^S25,S35,S99^  **Bonferroni & derivatives:**  Control the experiment-wise/type I error rate. ^S2,S5,S15,S16,S40,S43,S59,S63^ | **Alpha allocation/spending/recycling:**  **Bonferroni & derivatives:**  Can result in large sample size requirement if the hierarchy of endpoints is not considered (Bonferroni method can reduce power to detect differences on individual outcomes). ^S25,S65,S68^ Bonferroni is the least powerful (most conservative), followed by Holm’s, Hochberg’s, then Hommel’s tests. ^S5,S15,S59,S76,S99^ | **Bonferroni & derivatives:**  Can be incorporated with bootstrap methods to account for correlation ^S99^. Bonferroni can be conservative when testing multiple correlated endpoints. ^S2,S5,S15,S19,S40,S56,S59,S65,S68,S95,S100,S117^ |
|  | Scaled effect measures^S41^ |  |  |  |
|  | Approximate multinormal probabilities applied to correlated multiple endpoints^S37^ |  |  |  |
|  | New weighting scheme for Holm’s sequentially rejective a-adjustment procedure.^S119^ |  |  |  |
| **Global testing methods** | Method based on Global testing with estimation of correlation coefficient (for testing the primary and secondary endpoints) ^S25^ | Various global testing methods control type I error. ^S22,S30,S40,S48-50,S89,S121^  **IUT test:**  Controls type I error. ^S59,S64,S113^ No adjustment is required if all null hypotheses under the union are to be rejected. Otherwise, MTP is required. ^S56,S59^ | Various global testing methods have varying power. ^S11,S18,S20,S40,S48-50,S58,S59^ Can be used to enhance power/avoid loss of power due to individual testing and the power of the global test is larger than the power of each individual test if the assumption of common treatment effect is met. ^S2,S4,S22,S46,S48,S56,S68,S76,S95,S102,S108,S121^  **IUT test:**  Power can be reduced if all null hypotheses are to be rejected, hence larger sample size may be required. ^S56,S59,S64^ Power is at most equal to the minimum power of individual hypotheses. ^S113^ Power is affected by the degree of correlation between the outcomes. ^S44,S64,S87^ | Accounts for correlation between outcomes. ^S4,S95,S102^ Different global testing methods can suite MOCs with different degrees of correlation. ^S2,S6,S16,S30,S40,S50^  **IUT:**  Highly correlated outcomes can lead to increased power/reduced sample size depending on the effect sizes on the outcomes. ^S87,S113^ |
|  | Global test (modification of O'Brien's GLS) ^S69^ |  |  |  |
|  | Weighted rank-sum test^S49^ |  |  |  |
|  | Summation of Absolute value of Rank-based test (SAR) ^S50^ ***(NEW)^[[7]](#footnote-7)^*** |  |  |  |
|  | An extension of the methods of Hasler and Hothorn^S29^ |  |  |  |
|  | Extension of the Dunnett procedure to MOCs^S30^ |  |  |  |
|  | Risk score test^S20^ |  |  |  |
|  | A prediction-based test^S58^ ***(NEW)*** |  |  |  |
|  | A modified Intersection-Union Test^S67^ |  |  |  |
|  | Modification of OLS and GLS^S89^ |  |  |  |
|  | adaptations from O'Brien's rank-sum test for paired data with multiple outcomes^S40^ |  |  |  |
|  | A cluster-adjusted rank-based test^S121^ ***(NEW)*** |  |  |  |
|  | A global assessment procedure based on categorization of the individual endpoints to form an overall composite endpoint. ^S119^ |  |  |  |
| **Composite outcome related tests** | Composite outcome treatment heterogeneity test^S70^ **(*NEW)*** | Resolves the need for multiplicity adjustment. ^S14,S21,S64,S80,S99,S102,S117^ | Can be used to increase power for detecting treatment effects. ^S14,S21,S64,S65,S68,S70,S76,S79,S99^ Reduces sample size requirements due to increased power and event rate. ^S4,S14,S21,S77^ However, can result in loss of power if intervention effects on components are of different directions or sizes. ^S98,S102^ | Can be designed to include correlated components such as in benefit-risk assessment. ^S23,S70^ However, components are not essentially correlated. ^S4^ Different statistical tests for composite outcomes analysis can be applied for different directions of correlation. ^S6^ |
|  | A modification to win-ratio test^S7^ |  |  |  |
|  | A statistical test based on new composite hypotheses^S60^ |  |  |  |
| **Hierarchical, co-primary outcomes, and sequential tests** | Fractal Gatekeeping approach^S13^ | **Hierarchical method:**  Controls alpha in the strong sense. ^S13,S24^ Hypotheses are tested at full alpha according to prespecified order hence no adjustment is needed. ^S34,S59,S76^  **Sequential methods:**  Controls experiment-wise type I error. ^S5,S72^  **Co-primary endpoints:**  Requiring all hypotheses to be rejected resolves the need for multiplicity adjustment (testing to be at the full pre-specified alpha). ^S56,S59,S64,S76,S80,S84,S99^  **Gatekeeping procedures:**  Parallel gatekeeping controls type I error in the strong sense. ^S5,S86^ Serial gatekeeping resolves need for multiplicity adjustment. ^S56,S59,S99^ | **Sequential/hierarchical methods:**  Powerful due to the ability to use full alpha for the prespecified sequence of hypothesis testing ^S110^. Power is higher for hypotheses at the top of hierarchy and lower for the lower hypotheses. ^S99^  **Co-primary endpoints:**  Study power will depend on the degree of correlation between endpoints and the power to detect the required effect size on each. Hence study power can be less than the power of each individual co-primary endpoint. ^S56,S64^ |  |
|  | Hierarchical testing methods (stagewise & overall) ^S24^ |  |  |  |
|  | A generalized partially hierarchical test procedure in group sequential designs^S26^ |  |  |  |
|  | A flexible fixed-sequence testing method -an extension of Wiens’ approach^S36^ |  |  |  |
|  | A stepwise test with appropriate adjustment to the univariate p-values^S96^ |  |  |  |
|  | A method for upper bounds for type I error rates for testing key secondary outcome^S24^ |  |  |  |
|  | A greedy algorithm and a simulated annealing algorithm^S122^ |  |  |  |
| **Bayesian-related tests** | Optimal Bayesian design for Platform Trials with multiple endpoints (PMED) ^S31^ | Controls FWER ^S31^. Can also be combined with a frequentist approach to assess outcomes on a pre-specified alpha level^S64^  However, can be challenging to employ in practice and be overly subjective^S5^ | Robust and powerful. ^S31^  Can improve estimation efficiency. ^S5,S116^ | Can account for the dependency between outcomes. ^S64,S93^ |
|  | Bayesian approach to mixed treatment comparisons (MTCs) ^S32^ |  |  |  |
|  | Bayesian decision theoretic strategy^S82^ |  |  |  |
|  | A Bayesian strategy proposed by Thall, Simon and Estey - TSE strategy^S93^ |  |  |  |
|  | A Bayesian joint model ^S116^ |  |  |  |
| **Regression-based tests** | Scaled linear mixed model^S52^ | Can be difficult to interpret. ^S5^ | Efficient, powerful, can improve estimation efficiency. ^S27,S55,S91,S92,S123^ More powerful when outcomes are affected similarly. ^S52^ | Can accommodate correlation between outcomes. ^S3,S52,S91,S92,S102^ |
|  | Multivariate Multilevel Models^S3^ |  |  |  |
|  | Multivariate longitudinal potential outcome model (MCACE) ^S27^ |  |  |  |
|  | Generalized Mixed Effects Model and Transitional Models^S55^ |  |  |  |
|  | A reverse regression approach^S123^ |  |  |  |
|  | multivariate approach using a latent variable^S91^ |  |  |  |
| **Combined methods** | Graphical approach to Bonferroni-based closed test procedures^S12^ | Combination of tests can perform as good as or better than the original tests achieving strong control of type I error, however computation can be a concern in some situations. ^S48,S54,S73,S86,S96,S111^ While the combination can be developed to address MOCs in a specific setting, it may be possible to extend the combined test to other contexts with similar performance on type I error control. ^S88^ | Combining tests can be a way of increasing the power to detect treatment effects on individual endpoints and/or the global hypothesis and can increase the robustness of the results with minimal loss of power. ^S48,S54,S73,S75,S86,S88,S109^ | Some of the combined tests can perform better than its constituents in the case of highly correlated outcomes. ^S54^ Combination of tests can be done to accommodate dependence among MOCs. ^S75^ |
|  | Combining global and marginal tests^S54^ |  |  |  |
|  | Stratified multivariate Mann‐Whitney estimator combined with a closed testing procedure^S85^ |  |  |  |
|  | Closed testing procedures for group sequential clinical trials (combined with global test) ^S88^ |  |  |  |
|  | A global test followed by a closed testing procedure^S96^ |  |  |  |
|  | A fixed sequence Bonferroni procedure^S111^ |  |  |  |
|  | A Dunnett–Bonferroni-based parallel gatekeeping procedure^S114^ |  |  |  |
|  | A stepwise closed test procedure^S48^ |  |  |  |
|  | multi-way average closed testing under a parallel gatekeeping framework^S86^ |  |  |  |
|  | Resampling-based cut-off tests (bootstrap & cut-off combination) ^S75^ |  |  |  |
|  | Simes-Hommel intersection-union tests^S109^ |  |  |  |
|  | Resampling-Based intersection-union tests^S109^ |  |  |  |
|  | Bonferroni-closed test^S73^ |  |  |  |
|  | Hochberg-Bonferroni test^S73^ |  |  |  |
|  | Modified Hochberg- Bonferroni procedure^S73^ |  |  |  |
|  | Hochberg-closed test^S73^ |  |  |  |
|  | Closed testing procedure combined with other tests^S47^ |  |  |  |
|  | A closed procedure based on Follmann's test^S106^ |  |  |  |
| **Closed testing procedure** | Newman-Keuls MCP procedure (Closed stepwise approach) ^S8^ | This approach has good control of type I error in the strong sense. It can be incorporated with other tests, e.g. global testing, to yield conclusions on individual hypotheses. Can be used to test primary outcomes that has not been pre-specified. ^S8,S15,S43,S48,S59,S76,S99,S106,S108^  It can be difficult to handle with large number of individual hypotheses. ^S59^ | Closed testing procedures are powerful. ^S8,S48,S106^ |  |
|  | Bootstrap based method^S10^ |  |  |  |
| **Bootstrap tests** | A new partitioning testing strategy. ^S103^ | Can fail to detect global significance.^S16^ Can control the FWER.^S97^ | Improve power and can be efficient for sample size.^S9,S76,S99,S108^ Can be used to avoid loss of power in some situations. ^S5^ | Account for correlation.^S9,S59,S76,S97,S99,S108^ |

# Supplementary File 8

Results of the updated bibliographic databases search from 22 July 2022 to 16 January 2025.

## References of the updated database search

S8-1. Bishop DVM. Using multiple outcomes in intervention studies: improving power while controlling type I errors. *F1000Research*. 2023;10(no pagination)991.

S8-2. Chen J, Thakur M, Zeng H, Gabriel A. Modified truncated Hochberg procedure for multiple endpoints: An application in a confirmatory trial for pediatric functional constipation. *Contemporary Clinical Trials*. 01 Jun 2023;129(no pagination)107185.

S8-3. Chi X, Yuan Y, Yu Z, Lin R. A generalized calibrated Bayesian hierarchical modeling approach to basket trials with multiple endpoints. *Biometrical journal*. 01 Mar 2024;Biometrische Zeitschrift. 66(2):e2300122.

S8-4. Du Y, Li J, Raha S, Qu Y. A unified Bayesian framework for bias adjustment in multiple comparisons from clinical trials. *Statistics in Medicine*. 10 Jul 2024;43(15):2928-2943.

S8-5. Gao P, Li Y. Adaptive Multiple Comparison Sequential Design (AMCSD) for clinical trials. *Journal of Biopharmaceutical Statistics*. 2024;34(3):424-440.

S8-6. Hunter KB, Miratrix L, Porter K. PUMP: Estimating Power, Minimum Detectable Effect Size, and Sample Size When Adjusting for Multiple Outcomes in Multi-Level Experiments. Article. *Journal of Statistical Software*. Feb 2024;108(6):1-43. doi:10.18637/jss.v108.i06

S8-7. Kim H, Shahbal H, Parpia S, et al. Trials using composite outcomes neglect the presence of competing risks: a methodological survey of cardiovascular studies. *Journal of Clinical Epidemiology*. 01 Aug 2023;160:1-13.

S8-8. Luo X, Quan H. Some Multiplicity Adjustment Procedures for Clinical Trials with Sequential Design and Multiple Endpoints. *Statistics in Biopharmaceutical Research*. 2024;16(1):104-115.

S8-9. Mulier G, Chevret S, Lin R, Biard L. Bayesian Optimal Designs for Multi-Arm Multi-Stage Phase II Randomized Clinical Trials with Multiple Endpoints. Article. *Statistics in Biopharmaceutical Research*. Jul 2 2024;16(3):315-325. doi:10.1080/19466315.2024.2344543

S8-10. Montgomery RN, Ptomey LT, Mahnken JD. A flexible test for early-stage studies with multiple endpoints. *J Appl Stat*. 2023;50(15):3048-3061. doi:10.1080/02664763.2022.2097204

S8-11. Ordak M. Multiple comparisons and effect size: Statistical recommendations for authors planning to submit an article to Allergy. *Allergy: European Journal of Allergy and Clinical Immunology*. 01 May 2023;78(5):1145-1147.

S8-12. Parker RA, Weir CJ. Multiple secondary outcome analyses: precise interpretation is important. *Trials*. 2022/01/10 2022;23(1):27. doi:10.1186/s13063-021-05975-2

S8-13. Prunas O, Willemsen JE, Bont L, Pitzer VE, Warren JL, Weinberger DM. Incorporating Data from Multiple Endpoints in the Analysis of Clinical Trials: Example from RSV Vaccines. *Epidemiology*. Jan 1 2024;35(1):103-112. doi:10.1097/ede.0000000000001680

S8-14. Prunas O, Willemsen J, Warren JL, et al. Workshop on the design and use of clinical trials with multiple endpoints, with a focus on prevention of RSV. *Vaccine: X*. 01 Aug 2024;19(no pagination)100509.

S8-15. Ramírez PC, Diaz-Quijano FA. Bias attributable to the use of a composite outcome in evaluating a cocoa extract supplement. *Am J Clin Nutr*. Nov 2022;116(5):1452. doi:10.1093/ajcn/nqac185

S8-16. Seifu Y, Mt-Isa S, Duke K, et al. Design of paediatric trials with benefit-risk endpoints using a composite score of adverse events of interest (AEI) and win-statistics. *Journal of Biopharmaceutical Statistics*. 2023;33(6):696-707.

S8-17. Stringer D, Payne M, Carter B, Emsley R. The analysis and reporting of multiple outcomes in mental health trials: a methodological systematic review. *BMC medical research methodology*. 21 Dec 2024;24(1):317.

S8-18. Walker HGM, Brown AJ, Vaz IP, et al. Composite outcome measures in high-impact critical care randomised controlled trials: a systematic review. *Critical Care*. 01 Dec 2024;28(1) (no pagination)184.

S8-19. Watson SI, Akinyemi JO, Hemming K. Permutation-based multiple testing corrections for -values and confidence intervals for cluster randomized trials. *Statistics in Medicine*. 2023;42(21):3786-3803. doi:https://doi.org/10.1002/sim.9831

S8-20. Wen J, Hu C, Wang MC. Joint inference for competing risks data using multiple endpoints. *Biometrics*. 01 Sep 2023;79(3):1635-1645.

S8-21. Yuki S, Tanioka K, Yadohisa H. Estimation and visualization of heterogeneous treatment effects for multiple outcomes. *Statistics in Medicine*. 28 Feb 2023;42(5):693-715.

S8-22. Zhao B, Fine J, Ivanova A. Finding the best subgroup with differential treatment effect with multiple outcomes. *Statistics in Medicine*. 15 Jun 2024;43(13):2487-2500.

S8-23. Zou G, Zou L. A Nonparametric Global Win Probability Approach to the Analysis and Sizing of Randomized Controlled Trials With Multiple Endpoints of Different Scales and Missing Data: Beyond O'Brien-Wei-Lachin. *Statistics in Medicine*. 10 Dec 2024;43(28):5366-5379.

S8-24. Zhan T, Zhou Y, Geng Z, et al. Deep historical borrowing framework to prospectively and simultaneously synthesize control information in confirmatory clinical trials with multiple endpoints. *Journal of Biopharmaceutical Statistics*. 2022;32(1):90-106.

S8-25. Zhang Z, Lin Y, Liu J. Probability of Study Success (PrSS) Evaluation Based on Multiple Endpoints in Late Phase Oncology Drug Development. *Statistics in Biopharmaceutical Research*. 2023;15(3):675-688.

## Table 8-1: Characteristics of the articles identified from the updated search (July 2022-Jan 2025).

| **1st Author** | **Publication Year** | **1st author country** | **Affiliation** | **Publication type** | **Research area** | **Description of Specific research area** | **Funding** |
| --- | --- | --- | --- | --- | --- | --- | --- |
| Bishop, D. ^S8-1^ | 2023 | UK | University | Journal article | General statistical method |  | No funding |
| Chen, J. ^S8-2^ | 2023 | US | Pharmaceutical company | Journal article | Specific research area | Phase III trials with MOCs and multiple doses^[[8]](#footnote-8)^ | Pharmaceutical company |
| Chi, X. ^S8-3^ | 2024 | US | University | Journal article | Specific research area | Basket trials | Public research funding |
| Du, Y. ^S8-4^ | 2024 | US | Pharmaceutical company | Journal article | General statistical method |  | Funding not reported |
| Gao, P. ^S8-5^ | 2024 | US | Consultancy | Journal article | Specific research area | Adaptive trials (multi-arm multi-stage trials) | No funding |
| Hunter, K. ^S8-6^ | 2024 | Australia | University | Journal article | Specific research area | Multi-level experiments. |  |
| Kim, H. ^S8-7^ | 2023 | Canada | University | Journal article | Specific research area | Cardiovascular research | No funding |
| Luo, X. ^S8-8^ | 2024 | US | Pharmaceutical | Journal article | Specific research area | Multi-stage trials | Funding not reported |
| Mulier, G. ^S8-9^ | 2024 | France | Research institute | Journal article | Specific research area | Adaptive trials (Multi-arm multi-stage Phase II RCTs)^[[9]](#footnote-9)^ | Partially public research funding |
| Montgomery, R. ^S8-10^ | 2023 | US | Academic Hospital | Journal article | Specific research area | Early-stage trials | Mixed funding sources |
| Ordak, M. ^S8-11^ | 2023 | Poland | University | Editorial | General guidance |  | No funding |
| Parker, R. ^S8-12^ | 2022 | UK | University | Journal article | General guidance |  | No funding |
| Prunas, O. ^S8-13^ | 2024 | Switzerland | University & research institute | Journal article | Specific research area | Vaccine trials | Mixed funding sources |
| Prunas, O. ^S8-14^ | 2024 | Switzerland | University & research institute | Workshop summary | Specific research area | Vaccine trials | Non-profit organisation |
| Ramírez, P. ^S8-15^ | 2022 | Colombia | University | Letter to editor | General guidance |  | Public research funding |
| Seifu, Y. ^S8-16^ | 2023 | US | Pharmaceutical | Journal article | Specific research area | Trials with Benefit/risk  analyses | No funding |
| Stringer, D. ^S8-17^ | 2024 | UK | University | Journal article | Specific research area | Mental health | Mixed funding sources |
| Walker, H. ^S8-18^ | 2024 | Australia | Hospital | Journal article | Specific research area | Critical care trials | No funding |
| Watson, S. ^S8-19^ | 2023 | UK | University | Journal article | Specific research area | Cluster randomised trials |  |
| Wen, J. ^S8-20^ | 2023 | US | University | Journal article | Specific research area | Competing risk data | Public research funding |
| Yuki, S. ^S8-21^ | 2023 | Japan | University | Journal article | Specific research area | Heterogenous treatment effects | Public research funding |
| Zhao, B. ^S8-22^ | 2024 | US | University | Journal article | Specific research area | Heterogenous treatment effects | Public research funding |
| Zou, G. ^S8-23^ | 2024 | Canada | University | Journal article | Specific research area | MOCs of various scales |  |
| Zhan, T. ^S8-24^ | 2022 | US | Pharmaceutical company | Journal article | General statistical method |  | Mixed funding sources |
| Zhang, Z. ^S8-25^ | 2023 | US | Pharmaceutical | Journal article | Specific research area | Phase II/III trials | Funding not reported |

## Table 8-2: Statistical methods identified from the updated search (July 2022-Jan 2025).

| **The identified method** | **Brief description** | **Pros** | **Cons** |
| --- | --- | --- | --- |
| Two-stage Deep Neural Network (DNN)-guided algorithm^S8-24^ | A method based on Bayesian Hierarchical model; to approximate posterior probabilities and estimate critical values by deep learning for pre-specified strategies of hypothesis testing. | - Strong control of Type I error.  - Can accommodate varying degrees of correlation.  - Can be appealing to regulatory agencies due to prospective nature.  - Less computational time in comparison to methods in comparison. | - May require more time to simulate training data for the algorithm. |
| Adaptation from Bayesian Optimal design for Phase II clinical trial (BOP2) for MOCs^[[10]](#footnote-10)S8-9^ | A Bayesian design method for Type error control in multi-arm multi-stage Phase II trials with MOCs measuring efficacy and toxicity simultaneously. | - Can be robust in situations of deviation from planned design/methods.  - Optimised to control Type I error in situations where all arms assumed to be ineffective and toxic.  - Can accommodate correlation between efficacy and toxicity outcomes | - Setting stringent boundaries for remaining unpromising trial arms can gain power for the promising arms; however, this may result in higher type I error. |
| A modified truncated Hochberg procedure with a fixed sequence hierarchical testing procedure^S8-2^ | A method to control FWER in confirmatory phase III trials with MOCs and multiple doses.^[[11]](#footnote-11)^ | - More powerful than the original truncated Hochberg procedure. | - Less flexible than original truncated Hochberg procedure. |
| BAMBOO method (BAyesian Model that Brings phase 2 cOmposite endpOints) ^S8-25^ | A method to calculate the Probability of success (PrSS) of a phase III trial from phase II trial with MOCs. The method is applied to cancer trials with progression-free survival and overall survival as MOCs. | - Correlation can be calculated via meta-analysis of historical data or be simulated.  - Due to it’s Bayesian nature, it can be combined with other approaches to tackle practical challenges of oncological trials design/analyses. |  |
| PUMP (Power Under Multiplicity Project) ^S8-6^ | A tool for estimation of power, sample size, minimal detectable effect size in the context of multi-level RCTs with MOCs. R package is provided. | The tool accommodates MTPs controlling FWER or FDR, accommodate MTPs with various accountability of MOCs correlation.^[[12]](#footnote-12)^ |  |
| A method extended from Hochberg’s, Hommel’s, and Graphical procedures for sequential testing^S8-8^ | The method targets sequential multi-stage trials with to strongly control type I error for MOCs using alpha spending function for each endpoint. | - The proposed method may have higher overall power or rejecting probability compared with the original graphical procedure.  - Flexible as each stage can have a separate alpha-spending function. | Can be more computational given the sequential design and simulations required.  Further research needed to determine optimal alpha spending function for each endpoint in this setting. |
| Modifications to prediction-based procedure for MOCs^S8-10^ | The prediction test was previously proposed, and the paper introduces measures to address limitations of the prediction test using bootstrap methods to estimate the null distribution. | - The improved test is more powerful and less conservative than the original prediction test. | -Correlation between MOCs need to be estimated.  -Difficulties of interpretation of testing procedures applies to this method. |
| Regression-based method for MOCs of different scales (Global Win Probability) ^S8-23^ | A Nonparametric approach for handling MOCs of different scales. Sample size calculations and confidence interval estimation is incorporated with a review of the test performance with missing data. | - Robust with data missing at completely at random or at random.  - can be applied using standard statistical software. |  |
| Extensions to calibrated Bayesian hierarchical modelling^S8-3^ | The method aims for monitoring phase II basket trials with MOCs. | - Can be provide better type I error control and power than methods in comparison and reduce sample size requirement.  - Flexible procedures with various outcome types. | Does not perform as well when an outcome is not observed or missing, alternative multiple imputation procedures are proposed. |
| A framework for estimation of heterogenous treatment effects on MOCs^S8-21^ | The method uses weighting approach and latent variables to estimate heterogenous treatment effects on binary and continuous MOCs, in addition to identification of relevant subgroups for which the treatment may be more relevant. | - More accurate than methods in comparison.  Easy interpretation of subgroups due to visualisation method proposed. | The method assumes mutual independence for binary outcomes, further research suggested. |
| A permutation-based test with weighted averages of individual outcomes (wavP) ^S8-13^ | The method pools test statistics of individual outcomes together using inverse variance weighted average of relative risks, in the context of vaccine trials. Different scenarios based on effect size and incidence are explored and compared to other MOCs analysis procedures. | - The method has more power than procedures in comparison where effect sizes on outcomes were comparable, while controlling for type I error. | Can be challenging where treatment arm has zero occurrences on an outcome; however, a solution is proposed by authors. |
| Joint multiple cumulative incidence functions (CIF) procedure^S8-20^ | A method for addressing competing risk data arising in trials with MOCs. The focus is on events where ending statuses have different clinical interpretations or simultaneous relevance. | - Can reduce sample size and increase power.  - Useful when competing risk events have opposing clinical relevance.  - Can be adapted for composite outcomes |  |
| Permutation-based extensions of MTPs for cluster randomised trials^S8-19^ | Permutation-based adaptations of common MTPs for p-value adjustment with provision of a novel method for estimation of adjusted confidence intervals in the context of cluster randomised trials with MOCs. | Methods can facilitate power estimation via simulation. |  |

## Table 8-3: A summary of guidance on handling MOCs from articles identified from the updated search (July 2022-Jan 2025).

| **Reference** | **Focus** | **New themes** |
| --- | --- | --- |
| **Bishop, D. ^S8-1^** | Reviewing the use of a pre-existing statistical method (MEff), commonly used in genetics research, to the settings of trials with MOCs with considerations of power gain particularly for correlated outcomes. | No new relevant themes. |
| **Du, Y. et al. ^S8-4^** | A unified Bayesian framework to adjust for selection bias in trials with multiple comparisons (including MOCs multiplicity). The article discusses the challenges of multiple comparisons (including MOCs multiplicity) as a source of selection bias and how the proposed method can be routinely implemented in trials to reduce investments in development triggered by selection bias.^[[13]](#footnote-13)^ | **MOCs special application:** selection bias handling method for trials with MOCs. |
| **Gao, P. and Li, Y. ^S8-5^** | A method for sequential testing in multi-arm multi-stage trials that account for multiplicity sources including MOCs. the method includes sample size and power calculation, alongside sample-size re-estimation and inference estimates generation (p-values, confidence intervals, and point estimates). Advantages, disadvantages, and challenges of multi-arm multi-stage trials are discussed. | **MOCs special application:** multi-arm multi-stage trials with MOCs |
| **Kim, H. et al. ^S8-7^** | A review of cardiovascular trials on the use of competing risk analysis for trials with composite outcomes. The article explores reasons for limited use of competing risk analysis, overview of some relevant methods, and provide recommendations for improvement. | **Challenge**: lack of proper competing risk handling in the context of composite outcomes in cardiovascular trials.  No other new themes. |
| **Ordak, M. ^S8-11^** | Journal recommendations for authors on handling multiple comparisons. Focused on the need for appropriate post-hoc test selection and adjustment for multiplicity alongside the importance of reporting effect sizes and determination of minimal clinically important difference (MCID) for judging clinical relevance of results. | No new relevant themes |
| **Parker, R. and Weir, C. ^S8-12^^[[14]](#footnote-14)^** | Proposing careful and precise interpretations for unadjusted secondary outcomes showing strong statistical significance based on per-comparison error rate (PCER) that is not influenced by number of secondary outcomes. | Reporting secondary outcomes after the primary achieves significance, based on the PCER. |
| **Prunas, O. et al. ^S8-14^** | A summary from workshop discussing MOCs, approaches to multiplicity, sequential trial design, historical data, and meta-regression in the context of vaccine trials, with a special focus on Respiratory Syncytial Virus. Challenges of MOCs in vaccine trials and areas for further research are discussed. | No new relevant themes. |
| **Ramírez, P and Diaz-Quijano, F. ^S8-15^** | A letter reviewing the analysis and interpretation of composite outcome result of a cardiovascular trial. | No new relevant themes. |
| **Seifu, Y. et al. ^S8-16^** | A method to design trials evaluating treatments benefits and risks using a prioritized composite of efficacy measures and a composite score of Adverse Events of Interest (AEI), applying Win Ratio test for the analyses. Simulations include methods to estimate power and sample size for such a design. | **MOCs special application:** trials evaluating benefit and risk. |
| **Stringer, D. et al. ^S8-17^** | A review of MOCs use, handling, and reporting in mental health trials. | No new relevant themes. |
| **Walker, H. et al. ^S8-18^** | A review of composite outcome use, analysis, reporting, and challenges in critical care trials. | **Challenge**: due to heterogeneity of critical care syndromes, using composite can reduce sample size without necessarily increase probability of a statistically significant result.  No other new themes. |
| **Zhao, B. ^S8-22^** | Authors propose an extension method for identification and definition of the best subgroup for a treatment under investigation in the context of MOCs incorporating the treatment effect weight on each of the outcomes. The method is applicable to binary, continuous, and time-to-event outcomes. | **MOCs Special application**: The article provides overview of methods for definition and identification of subgroups and its challenges.  No other new relevant theme. |

.

1. The results on this table reflect findings from articles identified in the original database searches. [↑](#footnote-ref-1)
2. MTP: multiplicity testing procedure. [↑](#footnote-ref-2)
3. MACE: Major Adverse Clinical Outcomes, CHD: Coronary Heart Disease. [↑](#footnote-ref-3)
4. CONSORT: Consolidated Standards for Reporting Trials. [↑](#footnote-ref-4)
5. CTP: Closed-testing procedure, GSD: group sequential design. [↑](#footnote-ref-5)
6. The results on this table reflect findings from articles identified in the original database searches. [↑](#footnote-ref-6)
7. (NEW) refers to newly developed procedures. [↑](#footnote-ref-7)
8. MOCs: Multiple outcomes [↑](#footnote-ref-8)
9. RCTs: Randomised controlled trials. [↑](#footnote-ref-9)
10. MOCs: Multiple outcomes [↑](#footnote-ref-10)
11. FWER: Family-wise error rate. [↑](#footnote-ref-11)
12. MTPs: Multiplicity testing procedures, FDR: False discovery rate. [↑](#footnote-ref-12)
13. MOCs: Multiple outcomes. [↑](#footnote-ref-13)
14. This paper was identified through expert consultation. [↑](#footnote-ref-14)
